# Open-Source Joystick Manipulandum for Decision-Making, Reaching, and Motor Control Studies in Mice

**DOI:** 10.1523/ENEURO.0523-19.2020

**Published:** 2020-03-24

**Authors:** Parley P. Belsey, Mark A. Nicholas, Eric A. Yttri

**Affiliations:** 1 Department of Biological Sciences, Carnegie Mellon University, Pittsburgh, PA 15213; 2 Carnegie Mellon Neuroscience Institute, Carnegie Mellon University, Pittsburgh, PA 15213

**Keywords:** decision-making, high-throughput, mouse, operant, reaching, rodent

## Abstract

To make full use of optogenetic and molecular techniques in the study of motor control, rich behavioral paradigms for rodents must rise to the same level of sophistication and applicability. We describe the layout, construction, use and analysis of data from joystick-based reaching in a head-fixed mouse. The step-by-step guide is designed for both experienced rodent motor labs and new groups looking to enter into this research space. Using this platform, mice learn to consistently perform large, easily-quantified reaches, including during a two-armed bandit probabilistic learning task. The metrics of performance (reach trajectory, amplitude, speed, duration, and inter-reach interval) can be used to quantify behavior or administer stimulation in closed loop with behavior. We provide a highly customizable, low cost and reproducible open-source behavior training platform for studying motor control, decision-making, and reaching reaction time. The development of this software and hardware platform enables behavioral work to complement recent advances in rodents, while remaining accessible to smaller institutions and labs, thus providing a high-throughput method to study unexplored features of action selection, motivation, and value-based decisions.

## Visual Abstract

**Figure:**
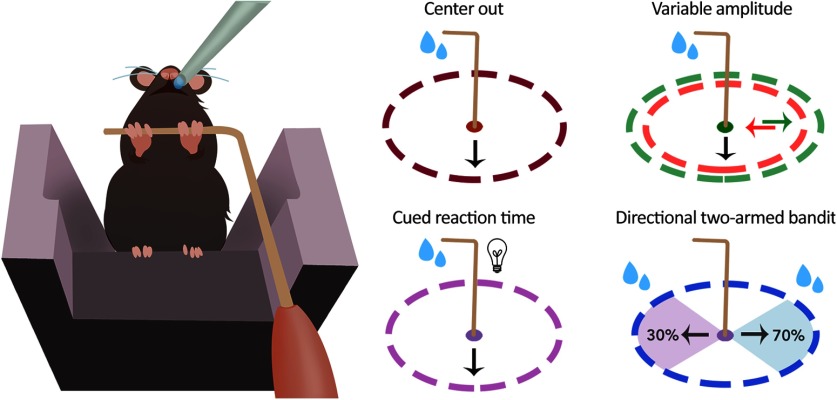

## Significance Statement

We are realizing that the behavioral repertoire of mice is much richer than previously thought, including motor control and decision-making using reaches. Modern neuroscience is now capturing this richness, paired with new genetic tools, to understand fundamental neuroscience principles. Here, we provide an illustrated build guide, code, multiple use scenarios, and analytic tools to a low-cost, highly customizable mouse joystick. This tool will enable improved throughput, accessibility, and experimental design (e.g., spatiotemporal reach trajectories over lever presses) for labs wishing to study a range of reach-based experiments.

## Introduction

Reaching is a well-studied neuroscience paradigm, across several species (Fromm and Evarts, 1981; Churchland et al., 2012; Dean et al., 2012; Cherian et al., 2013; Yttri et al., 2013; Mathis et al., 2017). This goal-oriented movement is highly quantifiable, reproducible, and unitary, unlike other tasks common to rodents that require several actions, such as reorientation followed by locomotion across a cage (Tai et al., 2012; Lak et al., 2014). Despite its simplicity, the action provides rich spatiotemporal dynamics (Bollu et al., 2019) that do not exist in other paradigms such as lever presses. The quantification of such richness is made trivial through the use of joysticks. Joystick manipulandums have been used for decades in both human and nonhuman primates studies of reaching (Thoroughman and Shadmehr, 1999; Maeda et al., 2018), and more recently in rats (Slutzky et al., 2010). Because joysticks provide real-time readout of the *x* and *y* trajectory (and therefore position and speed information), joysticks enable the study of ongoing correlated neural activity (Paninski et al., 2004; Panigrahi et al., 2015) or stimulation in closed loop triggered off a specific spatiotemporal feature of movement (Yttri and Dudman, 2016). This quality presents a significant advantage over impressive, but *post hoc*, computer vision techniques (Guo et al., 2015; Mathis et al., 2018) that cannot yet offer real-time reporting of reach position.

The use of mice to study the spatiotemporal dynamics of behavior has increased considerably in recent years (Harvey et al., 2009; Cohen et al., 2015; Guo et al., 2015; Klaus et al., 2017). Through the application of genetic tools, unprecedented avenues of discovery have been made possible in the study of the brain, including those of decision-making and motor control. While considerable work using “center-out” reaching tasks have been done in human and non-human primates, performing similar studies in rodents provides many advantages. Beyond optogenetic manipulations, studying reaching movements in mice also supports high-throughput methods that rapidly accelerate our understanding of the underlying brain circuits. As a side-effect of this approach, researchers can better capture behavioral and animal variance (rather than the typical “two monkey” rule), while also greatly reducing monetary barriers to entry that may prohibit smaller labs and institutions from participating in behavioral work (Brunton et al., 2013; Guo et al., 2015). In order to take advantage of these features, however, expansion of rigorous mouse behavioral paradigms must occur (Fetsch, 2016). Here, an inexpensive, open source 2D joystick platform, including hardware, software, online and offline analysis code is described (https://github.com/YttriLab/Joystick). This joystick can be put into practice quickly and provides precise, millisecond resolution readouts of limb position in real-time. We describe its usage for a basic center-out task, a cued reaction time version of that task, and a bi-directional “two-armed bandit” probabilistic learning task.

## Materials and Methods

### Behavior rig hardware

**Figure 1.**
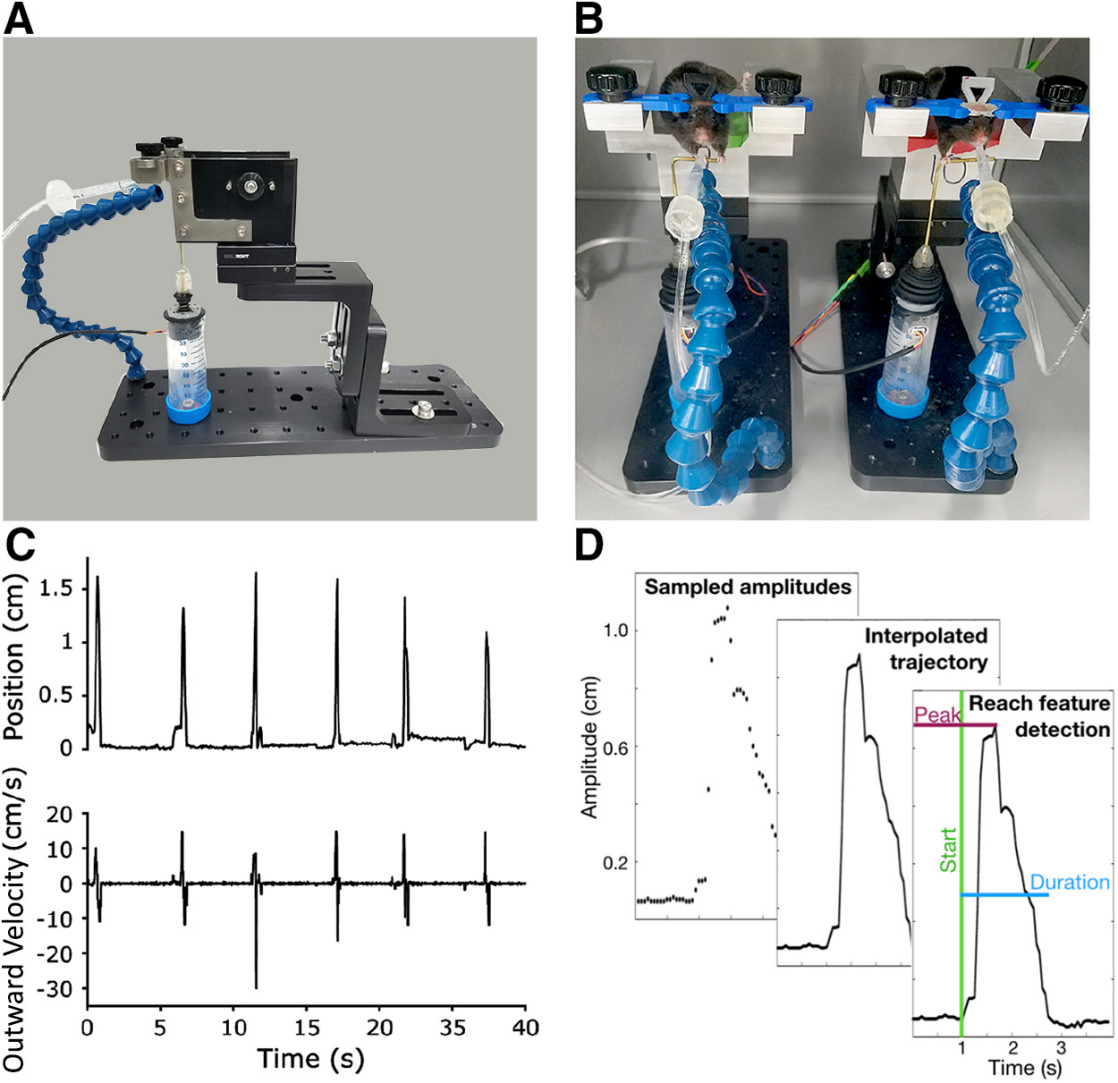
Reaching setup and performance. ***A***, Training platform hardware is adjustable and customizable to task demands. ***B***, Mice can perform reaching tasks in behavioral boxes and with minimal space usage. Reward water is dispensed through a solenoid circuit based on task parameters and mouse performance, monitored online with an Arduino. Joystick is positioned ∼2 cm directly below mouse’s eyes. ***C***, Amplitude (top) and outward velocity (bottom) traces from a trained mouse performing the basic center-out task. ***D***, Data processing flow for a sample reach. Measured joystick positions sampled over time are assembled into reach trajectories offline. The described software package identifies the reach start time and various features of each reach, including peak amplitude and duration.

Figure 1*A* shows a behavior rig (9” H × 4” W × 12” D), consisting of three main components: a removable head-fixation unit, joystick, and positionable sipping tube, all secured to an optical breadboard for easy arrangement. This setup works with a number of mouse head-fixation solutions; pictured in Figure 1*A* is the RIVETS system (Osborne and Dudman, 2014) and pictured in Figure 1*B* are custom built head-fixation units. The RIVETS system designs are available for download on the Dudman website on the Designs page, where the lab describes success using 3D printed and machined versions of the RIVETS system. The design for the custom platform unit is available to download on the Yttri Lab GitHub (https://github.com/YttriLab/Joystick/tree/master/Mouse%20Shuttle%20Parts). While the parts and plans for the shuttle-holding platform provide several advantages (solid construction, easy changing of height, automatic locking into place of the animal shuttle), the joystick platform should be amenable to most any head-fixation system. This animal shuttle is held in position with a precise yet easily removable knobbed magnetic base (Thorlabs, part KB2X2). The application of this piece in particular is a great function of the build, as it enables modularity and easy, never-fail docking of multiple head-fixed rigs.

The sipper tube is attached to Loc-Line tubing, allowing for easy positioning adjustments across animals. The water line and corresponding solenoid can be flushed using a 10% bleach solution followed by water for cleaning, with the Flush sketch available on the Yttri Lab GitHub. A spring-loaded, miniature hall effect joystick (Ruffy Controls, TS1) was chosen because it can relay the position of reaches with submillisecond delay and removes the potential for biases along the *x*- and *y*-axes that may be encountered with traditional two-axis potentiometer joysticks. The joystick is fixed at a height and distance that a head-fixed mouse can comfortably grasp the attached bar with both paws (∼2 cm below the mouse’s eye). The joystick can be mounted in a number of ways, including fitting into a 50 ml Falcon tube that is then screwed onto the breadboard, as shown in Figure 1*A*, *B*. This solution provides a surprisingly solid pedestal comparable to direct mounting to a more expensive, solid metal stand. Figure 1*B* demonstrates the rig’s small size and ease of assembly, which enables a lab to quickly set up dozens of rigs in a limited space.

While relatively inexpensive (Table 1), additional options to substantially reduce cost include a less substantial, permanent animal pedestal (could be directly bolted to surface, a savings of > $200) or using a two-axis potentiometer joystick (∼$5) in place of Hall effect joysticks (∼$75). The latter provides uniform resistance in every direction, instead of having two axes along which there is less resistance. These tracks have the potential to skew the 2D trajectory of the reach, although this may not be of consequence for some experimental questions. The moving parts of the potentiometers are also more likely to break down over time. If more delicate reach kinematics are of interest, we have observed that the Ruffy TS1 joystick resistance can be reduced by cutting the spring by up to 1.5 coils without risking the joystick’s ability to return to center. With one coil removed, it takes only 0.18 N to displace the joystick 1 cm. Other solutions include using a near zero resistance joystick designed for rodents, particularly that described in Bollu et al. (2019).

**Table 1:** Parts list for joystick build

Materials order form			
**Base Lab Tools**			
Item:	Catalog number:	Description:	Price:
¼ -20 stainless-steel screw kit	SKT25	Screw kit including ¼– 20 cap screw, set screws, nuts, washers	$49.00
Optical breadboard	SAB0412-D	4" × 12" × 1/2" solid aluminum optical breadboard, black anodized	$65.00
Right angle mounting bracket	ABS002	2" right angle mounting bracket, narrow slotted (1)	$39.00
**McMaster Carr**			
Item:	Catalog number:	Description:	Price:
0.34" ID, 0.875" OD washer	93490A030	Bronze washer for 5/16" screw size, 0.34" ID, 0.875" OD (pack of 10)	$5.70
1/16" ID, 1/8" OD tubing	6516T11	Tygon PVC clear tubing 1/16" ID, 1/8" OD, 25 ft length	$5.75
1/16" brass wire	8859K511	Ultra-formable 260 brass 1 foot long Rod, 1/16" diameter (pack of 3)	$2.03
Loc-Line	10095K97	Loc-Line coolant hose 1/4" trade size, female × male, 5 feet long	$28.20
Male Luer lock 1/8" ID hose barb	51525K291	Plastic quick-turn tube coupling Sockets, for 1/16" barbed tube ID, polypropylene (pack of 10)	$5.24
**Thorlabs**			
Item:	Catalog number:	Description:	Price:
Kinematic base	KB2X2	2" × 2" kinematic base, top and bottom plates, 1/4"-20 mounting	$83.39
**Ruffy Controls**			
Item:	Catalog number:	Description:	Price:
Miniature 2 axis hall effect joystick	TS1-1-R-R-1-BK	TS1, stepped cap, round limiter, rear mount, 0-5 v, black	$75.00
**Items from other retailors**			
Item:	Catalog Number:	Website:	Price:
3-Port Ported: Conventional Solenoid	LHDA1233115H	https://www.theleeco.com/products/electro-fluidic-systems/solenoid-valves/control-valves/lhd-series/3-port/ported/	$74.00
30-ml syringes	Description: 30-ml syringe Luer-Lok tip, box of 56	https://www.vitalitymedical.com/30-ml-syringes-without-needle.html	$25.84
50-ml conical tubes	14-432-22, case of 500	https://www.fishersci.com/shop/products/falcon-50ml-conicalcentrifuge-tubes-2/p-193321	$379.25
Battery clip connector	9-V clip on type battery snap connector lead wire plastic head, 10 pieces	https://www.amazon.com/Battery-Connector-Plastic-Atomic-Market/dp/B00IDHZ5FM	$6.99
1K diodes	1N5408 rectifier diode 3 A 1000 V	https://www.amazon.com/Parts-Express-1N5408-Rectifier-Diode/dp/B0009XSN02	$6.91
Electrical breadboard	BB400 solderless plug-in breadboard, 400 tie-points, 4 power rails, 3.3 × 2.2 × 0.3" (84 × 55 × 9 mm)	https://www.amazon.com/BB400-Solderless-Plug-BreadBoard-tiepoints/dp/B0040Z1ERO	$5.90
Elegoo board	Elegoo EL-CB-001 UNO R3 board ATmega328P ATMEGA16U2 with USB cable for Arduino	https://www.amazon.com/Elegoo-EL-CB-001-ATmega328PATMEGA16U2-Arduino/dp/B01EWOE0UU	$10.86
Heat shrink tubing, 1/8"	WindyNation 1/8" 20 Feet Black 3:1 Dual Wall Adhesive Glue Lined Marine Grade Heat Shrink Tube Tubing	https://www.amazon.com/WindyNation-20-Feet-Black-Adhesive/dp/B07LB8Z7HZ/ref=sr_1_4?keywords=1%2F8+heat+shrink+tubing&qid=1582813388&s=industrial&sr=1-4	$16.99
Jumper cables	40 pin male to female, 40 pin male to male, 40 pin female to female breadboard jumper wire ribbon Dupont cables kit, 120 pieces	https://www.amazon.com/COMeap-120pcs-Female-Breadboard-Jumper/dp/B01MU0IMFF/ref=sr_1_4?s=industrial&ie=UTF8&qid=1538699670&sr=1-4&keywords=male+male+jumper+cables	$7.99
Item:	Description:	Website:	Price:
LCD screen	LGDe home IIC/I2C/TWI LCD 1602 16 × 2 serial interface adapter module blue backlight for Arduino UNO R3 MEGA2560 (2 pack)	https://www.amazon.com/LGDehome-Interface-Adapter-Backlight-MEGA2560/dp/B0711WLVP9	$9.59
MicroSD card	MicroSD card 32GB,AUAMOZ microSDHC class 10 UHS-I high-speed memory card for phone, tablet, and PCs, with adapter (2 pack)	https://www.amazon.com/gp/product/B07DGHCFSM/ref=oh_aui_search_asin_title?ie=UTF8&psc=1	$16.12
MicroSD card reader for Arduino	SenMod 5PCS microSD card microSDHC mini TF card adapter reader module for Arduino	https://www.amazon.com/gp/product/B01JYNEX56/ref=ppx_yo_dt_b_asin_title_o00__o00_s00?ie=UTF8&psc=1	$8.29
RJ11 Telephone cable	C2G/cables to Go 02970 RJ11 modular telephone cable, silver (7 feet, 2.13 m)	https://www.amazon.com/C2GCables-Modular-Telephone-Silver/dp/B00006HSK6	$3.47
60-V transistors	Major brands TIP120. transistor, Darlington, NPN, 60 V, 5 A, 3-pin, 3+ Tab, TO-220, AmpB, rail, pack of 15	https://www.amazon.com/Major-Brands-TIP120-Transistor-Darlington/dp/B00B888622/ref=lp_306910011_1_7?s=industrial&ie=UTF8&qid=1538700353&sr=1-7	$5.99

Parts, prices, and vendors are included for the joystick build. Approximate cost of one setup: $440. PDF of step by step build instructions for creating the joystick training platform are available in Extended Data 1.

10.1523/ENEURO.0523-19.2020.ed1 Extended Data 1 Build manual for joystick training platform. Step by step build instructions and parts list for making the behavior rig described in Figure 1. Download Extended Data 1, PDF file.

The data acquisition hardware is comprised of an Arduino, solenoid circuit, microSD card reader, and LCD readout. Although not necessary for task execution, the LCD screen provides valuable information on animal performance and feedback during debugging. The uploaded Arduino sketch determines if a correct reach has been performed based on joystick position and timing and delivers the predetermined water reward. Real-time task information, such as number of reaches, time, number of punishments, and moving average of last five RTs are displayed on the LCD screen, and session data are written to a microSD card for saving and later analysis. This setup can also be used to deliver stimulation in closed loop with behavioral performance aspects (e.g., reach speed). Full build and part ordering instructions can be found at https://github.com/YttriLab/Joystick and (Extended Data 2).

10.1523/ENEURO.0523-19.2020.ed2 Extended Data 2 Online and offline joystick code. Online and offline code can be found at the Yttri Lab GitHub (https://github.com/YttriLab/Joystick). “Arduino Code” contains sketches to run the basic center-out reaching task, the VAO task, the reaction time task, and the directional-dependent two-armed bandit task. All tasks are capable of tracking real-time joystick position and allow for experimenter defined control of task parameters. The folder also includes code to flush fluid delivery lines for cleaning. Supplied in “Processing Code” is a sketch that can be used to visualize real-time joystick position as well as task performance and variables for the basic center-out reaching task. “MATLAB Code” contains the main offline analysis code (JSAnalysis.m) for the basic center-out reaching task, which collects data pertaining to task performance as well as reach kinematics. This folder also includes accessory analysis functions and a function (SavemicroSDData.m) to save and name data collected from the joystick. Download Extended Data 2, ZIP file.

Two methods of data acquisition were developed to satisfy a range experimental demands. Code written in Processing software displays a real-time visual of joystick position and task parameters while saving data to a .csv file. In addition to writing to a local machine, the data file can be written to a microSD card. Sending data wirelessly over Wi-Fi may also be performed. Other data relay methods, including solutions from LabJack or the Open Ephys acquisition system may be used with this joystick/Arduino build as well.

### Behavioral task software

Reach position is calculated through the Hall effect sensor in the joystick, which measures the magnitude of the magnetic field generated by magnet attached to the joystick, and output a proportionate voltage to the Arduino board (Stewart, 2017). From *xy* position output, the Arduino sketch calculates the Euclidean distance between “baseline” position and current joystick position. To complete a basic trial, mice perform bimanual reaches at a self-directed pace (Fig. 1*C*). The joystick setup can also be adapted to perform unimanual (or double unimanual) reaches. When the reach position surpasses the amplitude threshold, sweetened water is delivered after a 1-s delay. This delay is in place to help dissociate movement and reward representations in the brain. A new trial begins after a fixed, 3-s inter-trial interval (ITI), in order to obtain discrete movements. In our reaction time experiments, two adult C57/bl6 mice were used in each condition (one male, one female).

We describe the basic flow of data processing in Figure 1*D*. Code for running these tasks and offline data analysis used to quantify reach performance, including trajectory, amplitude, peak speed, duration, and inter-reach interval has been produced and is available at https://github.com/YttriLab/Joystick. Here, we also offer offline analysis code (Online and Offline Joystick Code available in Extended Data 2), although this study’s major contribution is in the form of the physical joystick design, construction, and online task code. In our offline analysis, reach detection is based on threshold crossing, and works forward and backwards in time from a minimal reach amplitude threshold crossing to determine exact reach initiation and termination times. In doing so, the user is able to select for only full reaches and ignore small “blips” due to postural adjustment, grooming, or other non-task related behavior.

**Figure 2.**
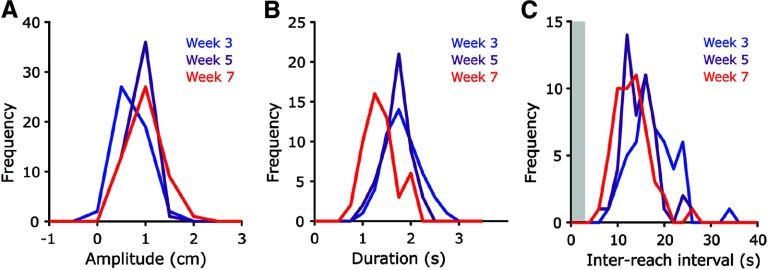
***A***, Peak amplitude, ***B***, reach duration, ***C***, inter-reach interval (IRI) for the first 50 trials performed in each session during weeks 3, 5, and 7. Inter-trial interval, shown in light grey, is set at 3 seconds in the basic task, and is included in IRI plot shown above.

## Results

Adult mice can be easily, and automatedly, trained to make large, reproducible reaches covering upwards of 2 cm. Figure 1*C* provides a demonstration of the online readout while the tool is in use. Video of mouse performance and online reach-position readout, including task state, threshold, time, and number of trials performed available in Movie 1. The joystick setup can capture fine variations in performance metrics including trajectory, outward velocity, amplitude, and duration (Fig. 2*A–C*).

Movie 1. Supplementary Joystick Performance and Readout. Video of mouse performance and online reach-position readout, including task state, threshold, time, and number of trials performed. 10.1523/ENEURO.0523-19.2020.video.1

For training, in addition to water control, we recommend the use of 3 mM acesulfame potassium, an artificial sweetener in the reward water. Artificial sweeteners are not readily digested by microbes, and thus require fewer line cleanings. This also eliminates potential concerns about the caloric quantity of the reward, though note that artificial sweeteners are perceived differently in the brain than sucrose (Kaskan et al. 2019). Prior to surgical implantation of the head cap, mice are exposed to experimenter handling. Two days after surgery, mice were head-fixed in the shuttle for increasing periods of time (5–45 min) and hand watered while head-fixed over the subsequent 3 d. Mice are placed on water control one to 3 d prior to the first day of experimentation and kept on water control for the duration of the experiment. Training is comprised of a two-week period where mice are fixed in the behavioral rig, in a darkened behavior box (18” H × 20” W × 22” D) for 30 min at a time (Fig. 1*B*). In the first 3 d, mice perform two, 30-min sessions per day (morning and afternoon) to increase the rate of learning of the reaching behavior, while single 30-min session are performed for the rest of training and experimentation. Over the course of the training period, threshold to receive water increases from 0.1 cm, where almost any movement of the joystick results in water reward, to 0.9 cm, with naive mice reaching expert level (defined as >100 successful reaches/session with a reward threshold of >0.9 cm from center) in two to three weeks. After the initial joystick movement-water connection is established (typically one to six sessions), set threshold is increased gradually (no more than 1 mm/d), as strength and endurance to complete the task needs to be built up. Delay to reward is increased gradually (50 ms/d) along with threshold from 500 ms to 1 s as mice reached 0.9-cm reach proficiency. As shown in Figure 2*A–C*, reach dynamics are refined after initial task learning has elapsed. All animal procedures were performed in accordance with the Carnegie Mellon University animal care committee’s regulations.

**Figure 3.**
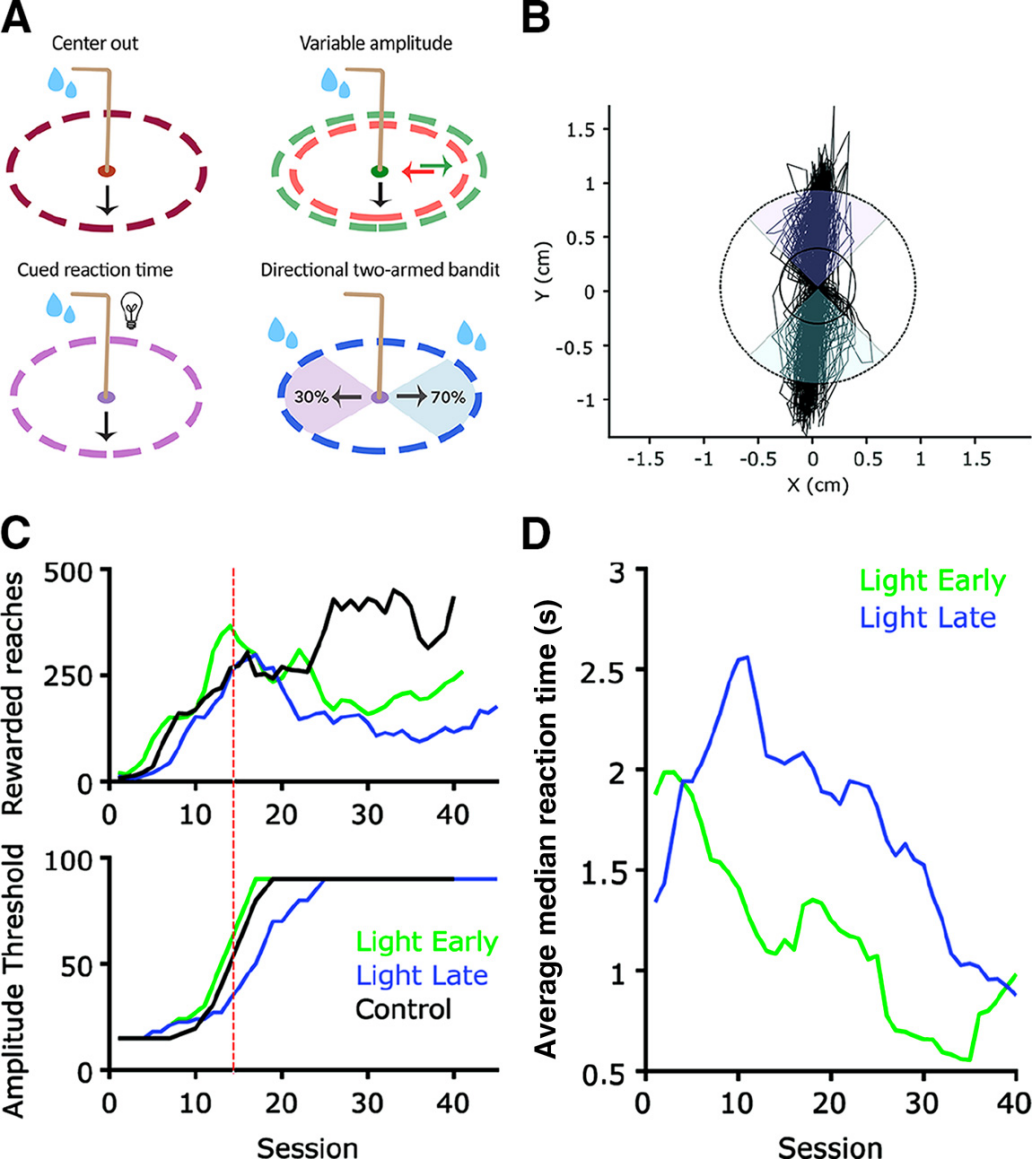
Training and performance of specialized reaching tasks. ***A***, The mouse joystick enables (1) a basic center-out task (all directions rewarded, dashed ring = reward amplitude threshold), (2) variable amplitude reaching wherein the reward amplitude can be changed within the session, (3) a reaction time task with a go-cue light, and (4) a bidirectional two-armed bandit task to assess decision-making, among other possibilities. In this case, reaches in different directions carry different reward probabilities. ***B***, To demonstrate the ability to make discrete reaches in two directions, we demonstrate an *x*-*y* joystick position trace over a 30 min, two-armed bandit task session wherein the high probability rewarded direction changed with throughout the task. The solid black circle denotes initial training threshold for rewarded reaches at 0.35 cm, and dashed circle representing expert level threshold of 0.9 cm. ***C***, Mean number of rewarded reaches (top) and reward threshold amplitude for each session performed by light early (green), light late (blue), and control (black) groups. ***D***, Average of median reaction times for each session during light early and light late groups, aligned to cued light introduction session.

While the variations on a reaching task are innumerable, several examples that reflect standard experiments common to the non-human primate literature in particular are provided (Fig. 3*A*). We provide code for the following: (1) basic center-out (direction agnostic) reaching task; (2) variable amplitude operant (VAO) task (Baraduc et al., 2013; Yttri and Dudman, 2018), in which the required threshold for reward is moved throughout the task; (3) reaction time version of the basic task, wherein a light provides a “go” cue; and (4) direction-dependent two-armed bandit task, in which a probabilistic reward contingency must be learned. Reaches in opposing directions carry different reward rates, and these rates change randomly (Fig. 3*B*).

We have found that it is best to start training on the desired task, rather than the basic task followed by later additions of complexity, hindered later learning of the tasks.

To further test the effectiveness of the joystick platform in conjunction with automated training efficacy, two training paradigms for a reaction time reach task using a light go-cue were tested. The timing of the introduction of the light cue was used as a dependent variable. In the “light early” condition, the go-cue light was introduced on the first day of training. The “light late” condition introduced the go-cue in the seventh session. In both conditions, “punishment” for early reaches (a 5000-ms time out period paired with house lights and restart of trial with new, random ITI) was introduced at day 14 to discourage anticipatory reaches (Fig. 3*C*, dashed red line, two-tailed *t* test). Two animals (one female, one male) were used in each cue condition. No sex differences were observed (*p* > 0.6). As the *n* is quite small in this proof of concept documentation, further comparative statistics are of little use. Therefore, error bars have been left out of plots. These data demonstrate that mice can learn the task, and the trends shown may be of use to experimenters. The performance of two control animals (mice performing the basic center-out task with no go-cue) are also shown where appropriate.

All mice were able to learn the task to criterion, defined as performing at least 100 reaches over 0.9 cm in 30 min. Most animals surpassed this standard easily, with the majority performing over 100 reaches by session 7 and all achieving expert level by three weeks (>100 successful reaches over the 0.9-cm amplitude threshold). However, we observed a numerical advantage in the use of the light early over the light late (Fig. 3*C*). The number of trials performed in each of the last four sessions was significant across all conditions (early vs late, *p* < 0.05; control vs either condition, *p* < 0.01; two-tailed *t* test). More importantly, we observed that reaction time to the go-cue light reduced more quickly in the light early training regimen (Fig. 3*D*). We defined reaction time as rewarded movement initiation time minus cue light on time (all reaction times >5 s were omitted). A steady decrease in reaction time over training sessions can be observed. Further work must be performed to assess the generalizability of these observations to a larger cohort of animals, but our proof of concept data demonstrate that (1) mice can learn a cued-reaction time reach task and (2) introducing the entire task at once is likely to be preferable to a progressive, piecemeal approach (Kuhlman et al., 2014; Hong et al., 2018).

## Discussion

This work documents an open-source, inexpensive joystick apparatus capable of millisecond and submillimeter resolution and real-time applications. We demonstrate the construction, use, optimization, and offline analysis of the data generated by this modular apparatus. This joystick can be used to study several classic reaching paradigms: a basic center-out reaching task, a cued reaction time version of that task, and a bi-directional, two-armed bandit probabilistic learning task. Perhaps of most use, the described tool setup be used in automated training, thus enabling high-throughput research methods, a critical avenue for the future of neuroscience. While one may glean how to build joystick rigs from other sources (Bollu et al., 2019), we provide the first documentation of a self-centering joystick with extensive online task code and offline analysis.

In a direct study of the application of this joystick setup, it is demonstrated that mice can reliably learn and reproduce the reaching behavior trained through the designed hardware and software platform. Mice can learn the basic reaching task in two to three weeks. The speed of training is a pronounced advantage over non-human primate studies, which can take months or even years. Although there are some performance attributes that mice are unlikely to ever be capable of, this study (reaction time task, two-armed bandit) and several others continue to narrow the gap between mouse and monkey behavior (Galiñanes et al., 2018; Bari et al., 2019; Stringer et al., 2019).

Looking toward the other end of the spectrum, the implementation of a joystick manipulanum instead of traditional lever-press setups in rodent behavioral work setups seems obvious. Consider pushing a child on a swing or drinking a cup of hot coffee: the manner in which those actions are performed far exceeds the selection of those actions in importance. A reduced, one-dimensional joystick affords fundamental measures of movement speed and amplitude with little to no extra effort. Beyond this, measures like speed and amplitude can be used to assess vigor, motivation, and confidence (Resulaj et al., 2009). These factors are vital in understanding the effects of neural or behavioral perturbations. Learning, not just of what to do but how, is also readily assessed. The compact and modular nature of the setup allows additional task-related devices (e.g., light cue, odor delivery, multiple unimanual joysticks) to be easily integrated into the same experimental setup, thus maximizing the experimental possibilities within one setup.

A limitation with the current design is that animals prefer forward/backward movements rather than left/right movements. It is possible that with some modifications, (differently shaped grip, lower joystick resistance laterally), an animal could move the joystick in all directions equally, opening up greater possibilities for complex tasks. Additionally, while reaches to threshold are consistently trainable and reproducible, mice do learn their own ways of completing the movement, including some that “rev up” (performing a very small reach in the opposite direction before the large reach). Another concern is the tendency for animals to perseverate in tasks reacquiring multiple response directions. This difficulty is common to many tasks with changing demands. To avoid this, we recommend introducing most of the task aspects early in training, and introducing any later changes slowly. For example, introducing punishment timeouts to discourage extraneous reaching after the first week of training to allows mice to better learn, but not give up on, the task before they reach full expert level.

Studying the neural correlates of behavior requires precise, oftentimes real-time measures of those actions. In designing this joystick platform, we have created a low cost and customizable alternative to traditional center out tasks involving non-human primates. The steps to implementation for the hardware, software, online and offline analysis are laid out. This setup takes advantage of the experimental advantages mice offer, including genetic tools and high-throughput automated training-while providing rich spatiotemporal dynamics of motor control, action selection, and decision-making.

## Synthesis

Reviewing Editor: Alexxai Kravitz, Washington University in St. Louis Decisions are customarily a result of the Reviewing Editor and the peer reviewers coming together and discussing their recommendations until a consensus is reached. When revisions are invited, a fact-based synthesis statement explaining their decision and outlining what is needed to prepare a revision will be listed below.

This manuscript was seen by two reviewers and myself, and all were very positive on it being a very useful device and strong contribution to the open-source methods track of eNeuro. There was appreciation for the work that went into this, and the supplemental documentation containing the build instructions. The paper is excellent and we had no major concerns. However we think the following comments would improve clarity of the manuscript. I anticipate these revisions should not take you longer than 2 weeks (although feel free to take longer if you need it):

1) A description of the 3D printed head-fixing setup in Figure 1B could be included - are those designs available for download? Is the 3D printed approach OK for the RIVETS system or is it better for these parts to be machined as in Figure 1A?

2) The paper could use a sentence to explain how mice are acclimated or otherwise accustomed to head fixing procedure and apparatus. How long does this take? Are there any special considerations?

3) The paper would benefit from more information on how the signals from the joystick are typically analyzed. They could show an example of the raw data from the joystick and how they process it. This can be high-level (example images of raw traces and processing flowchart).

4) They should include a sentence explaining how to clean/flush the solenoid and the device. Is that procedure included in the code?

5) Both reviewers were curious how much force the joystick takes to move - was this measured?

## Author Response

We thank the reviewers for their comments, and are very excited about the opportunity to share this tool and software. We address the reviewer comments below:

1) A description of the 3D printed head-fixing setup in Figure 1B could be included - are those designs available for download? Is the 3D printed approach OK for the RIVETS system or is it better for these parts to be machined as in Figure 1A?

We believe our joystick platform is amenable to any of the several head-fixation solutions available. The reviewers point out that we demonstrate two - the RIVETS and an in-house produced shuttle. We have included plans for the latter in supplemental and have added to the github repository. While the parts and plans for the shuttle-holding platform provide several advantages (solid construction, easy changing of height, automatic locking into place of the animal shuttle), the real delivery is the joystick and accompanying code.

We have added the following text:

“ This setup works with a number of mouse head-fixation solutions; pictured in Figure 2A is the RIVETS system (Osborne and Dudman, 2014) and pictured in 2B are custom built head fixation units. The RIVETS system designs are available for download on the Dudman website on the Designs page, where the lab describes success using 3D printed and machined versions of the RIVETS system. The design for the custom platform unit is available to download on the [redacted] lab Github. While the parts and plans for the shuttle-holding platform provide several advantages (solid construction, easy changing of height, automatic locking into place of the animal shuttle), the joystick platform should be amenable to most any head-fixation system. ”

2) The paper could use a sentence to explain how mice are acclimated or otherwise accustomed to head fixing procedure and apparatus. How long does this take? Are there any special considerations?

We have expanded the already existing explanation that was on lines 130-132:

“Prior to surgical implantation of the head cap, mice are exposed to experimenter handling. Two days after surgery, mice were head fixed in the shuttle for increasing periods of time (5 - 45 min) and hand watered while in head-fixed over the subsequent three days.”

3) The paper would benefit from more information on how the signals from the joystick are typically analyzed. They could show an example of the raw data from the joystick and how they process it. This can be high-level (example images of raw traces and processing flowchart).

We have the requested figure, included here, to address this point. There is actually quite little in terms of signal processing that we do. Joystick position is recorded on the Arduino; we then interpolate 1KHz resolution and apply our reach detection algorithm (located on the cited gitHub repository) to identify the start time of the reach. The accompanying figure legend reads:

Figure 2. Data processing flow for a sample reach. Measured joystick positions sampled over time are assembled into reach trajectories offline. The described software package identifies the reach start time and various features of each reach, including peak amplitude and duration.

We would like to ask the editor that, if accepted, we might recombine figure 1 into “setup” (original panels a-c plus new data-flow figure as 1D), and “results of animal training” (original panels e-f) into a new figure 2.

4) They should include a sentence explaining how to clean/flush the solenoid and the device. Is that procedure included in the code?

We have added the following text:

“The sipper tube is attached to Loc-Line tubing, allowing for easy positioning adjustments across animals. The water line and corresponding solenoid can be flushed a 10% bleach solution followed by water for cleaning, with the Flush script available on the [redacted] lab Github. ”

5) Both reviewers were curious how much force the joystick takes to move - was this measured?

We have added the following text:

“...we have observed that the Ruffy TS1 joystick resistance can be reduced by cutting the spring by up 1.5 coils without risking the joystick's ability to return to center. With one coil removed, it takes only 0.18N to displace the joystick 1 cm. ”
