# Supplementary material for: Open-Source Joystick Manipulandum for Decision-Making, Reaching, and Motor Control Studies in Mice
Source: eNeuro. 2020 Mar 24;7(2):ENEURO.0523-19.2020. doi: 10.1523/ENEURO.0523-19.2020 (PMC7131984; doi:10.1523/ENEURO.0523-19.2020)
Supplement: Extended Data 1 — Build manual for joystick training platform. Step by step build instructions and parts list for making the behavior rig described in Figure 1. Download Extended Data 1, PDF file. [file enu-eN-OTM-0523-19-s03.pdf]

Table of Contents

Material List………………………………………………………………………… 1- 2

Assembly

Headfixing Unit..…………………………………………………………….3 - 4

Joystick Stand………………………………………………………………… 5 - 6

Data Collection………………………………………………………… 7 - 8, 10

Notes……………………………………………………………….. 11

Water Delivery……………………………………………………………… 8 – 10

Materials Order Form………………………………………………………..12 -13

Materials List: Headfixing Unit and Joystick Module

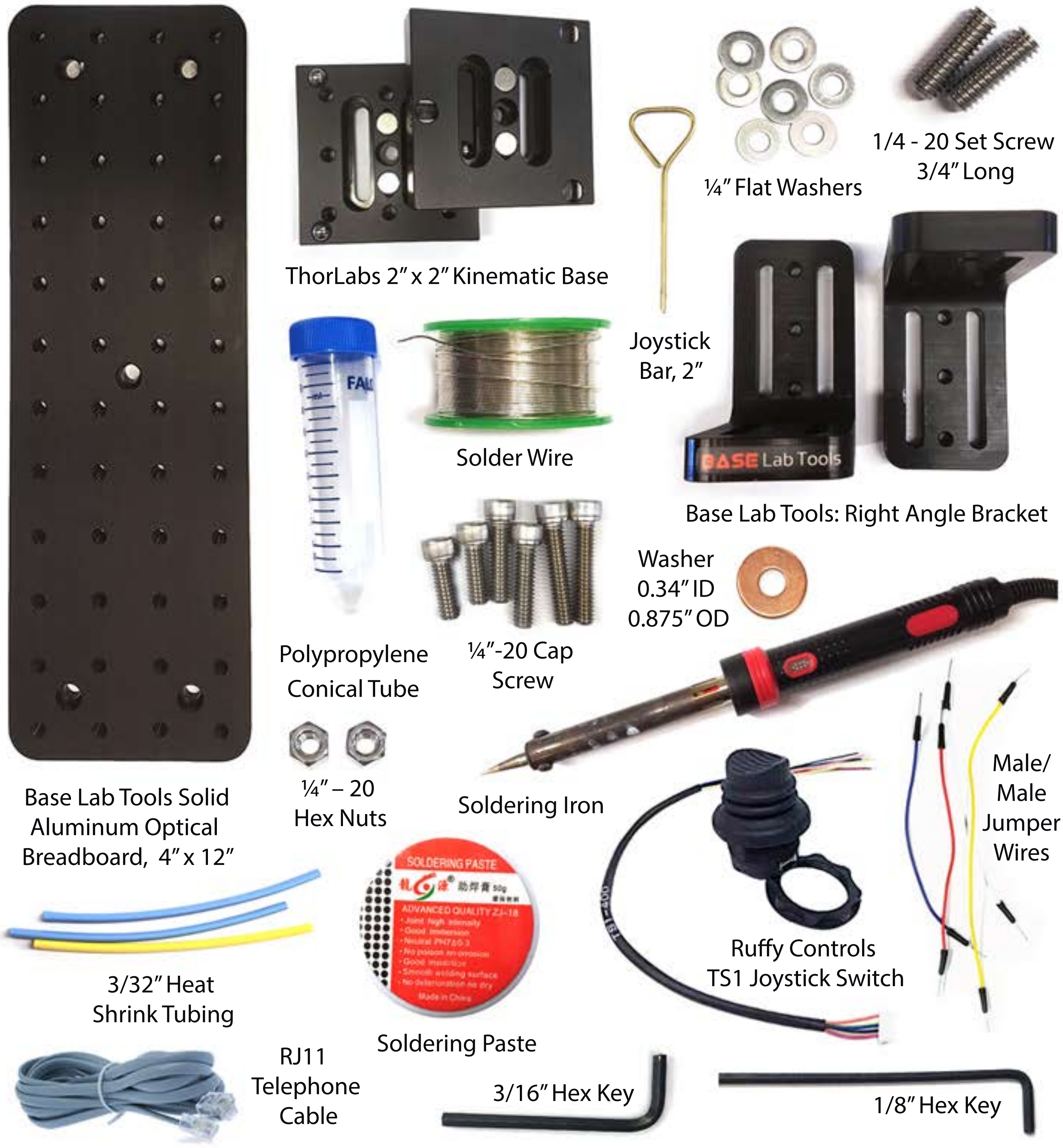

• 1, Polypropylene Conical Tube • 1, Base Lab Tools: Solid Aluminum Optical Breadboard, 4″ x 12″ • 2, Base Lab Tools: Right Angle Bracket, Narrow Slotted (ABS002) • 2, ¼″-20 Cap Screw, 1″ Long • 2, ¼″-20 Cap Screw, 1¼″ Long • 2, ¼″-20 Cap Screw, ¾″ Long • 7, ¼″ Flat Washers • 2, ¼″ – 20 Hex Nuts • 2, 1-4 -20 Set Screws, 3/4" Long • 1, Ruffy Controls TS1 series joystick unit • 4″, 1/16" Bronze Wire (for joystick bar) • Tape • 3/16″ Hex Key • 1/8″ Hex Key • Dental Acrylic, Resin and Liquid Monomer • Soldering Paste • Solder

Materials List: Data Collection and Water Delivery

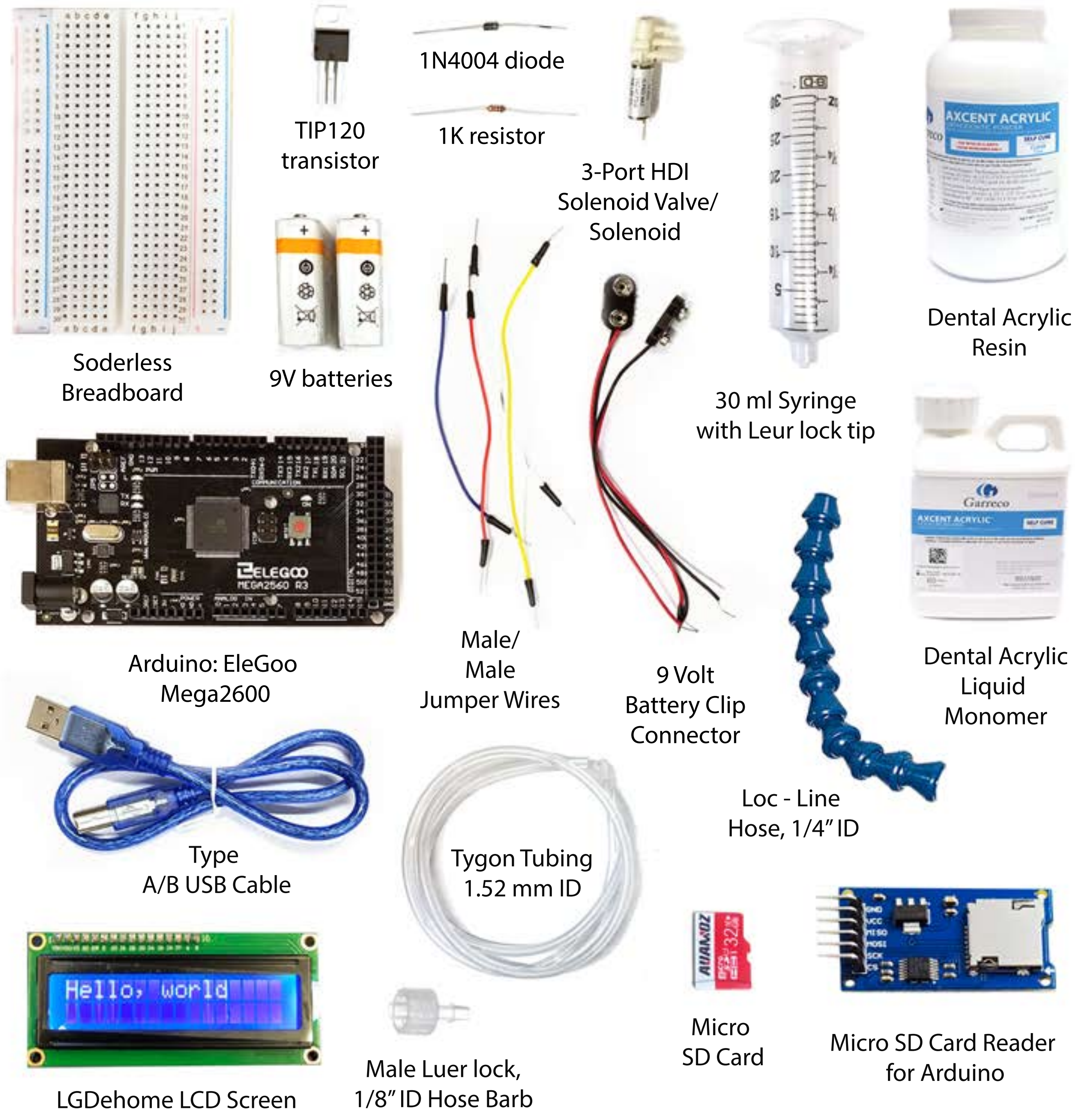

• 1, The Lee Co. 3-Port HDI Solenoid Valve (LHDA1231115H) • 2 lengths [to fit rig box], Tygon Tubing 1.52 mm ID • 1 Arduino board (EleGoo Mega2600) • 1,Type A/B USB Cable • 1, Soderless Breadboard • 2 Battery Clip Connectors • 1, 1K Resistor • 1, TIP120 transistor (or TIP102) • 1, 1N4004 diode (or 1N4001) • 2, 9V batteries • 8, Male/Male Jumper Wires • 6, Female/Male Jumper Wires • Loc - Line Modular Hose , 1/4” ID (apprx 15) • 1, LGDehome 16x2 Serial Interface Adapter for Arduino MEGA2560 • 1, Male Luer Lock, 1/8" ID Hose Barb • 1, Micro SD • 1, Micro SD Reader for Arduino • 30ml Syringe Sterile with Luer Lock Tip • Tape • Superglue • Dental Acrylic, Resin and Liquid Monomer

Goal: Build a headfixing device suitable for mice in electrophysiology experiments

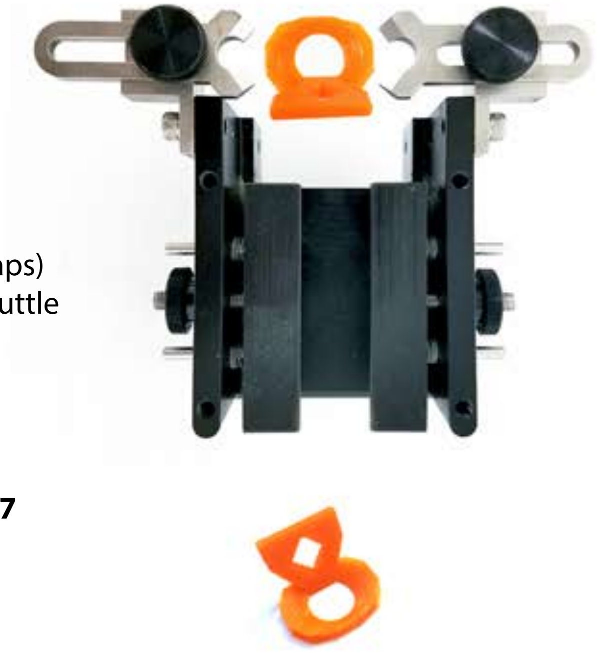

1. Using AutoCAD files and instructions found on:

http://dudmanlab.org/html/rivets_designs.html

manufacture RIVETs stystem parts (forks, desired head caps) and shuttle components. Assemble into an adjustable shuttle with headfixing capabilities.

2. Based on the Methods section of:

Osborne & Dudman (2014) PLoS One 9(2): e89007

permanently attach head caps to experimental mice.

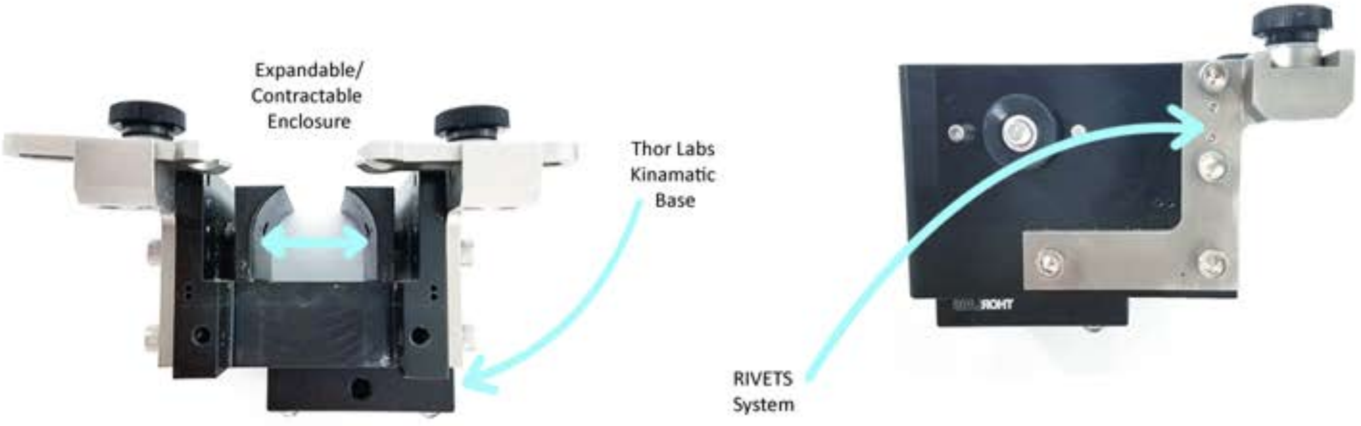

Goal: Build base and stand for headfixing shuttle.

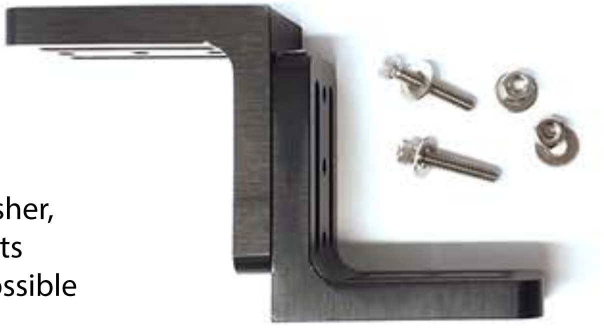

Steps:

1. Hold 2 right angle brackets in a "Z" shape, so that the slots align. Thread a 1 1/4" long, 1/4" - 20 cap screw with a 1/4" flat washer, and push through both bracket slots.

2. Cap the emerging end of the screw with a 1/4" flat washer, and then a 1/4" - 20 hex nut in order to hold the 2 brackets together. Join the pieces at the lowest extension level possible so adjustments can be made in later steps.

3. Repeat steps 1 and 2 with the other slot for security. In order to fully tighten hex nuts onto the screws hold the cap of the scew in place with a 3/16" hex key, and tighten the nut with a wrench or pliers.

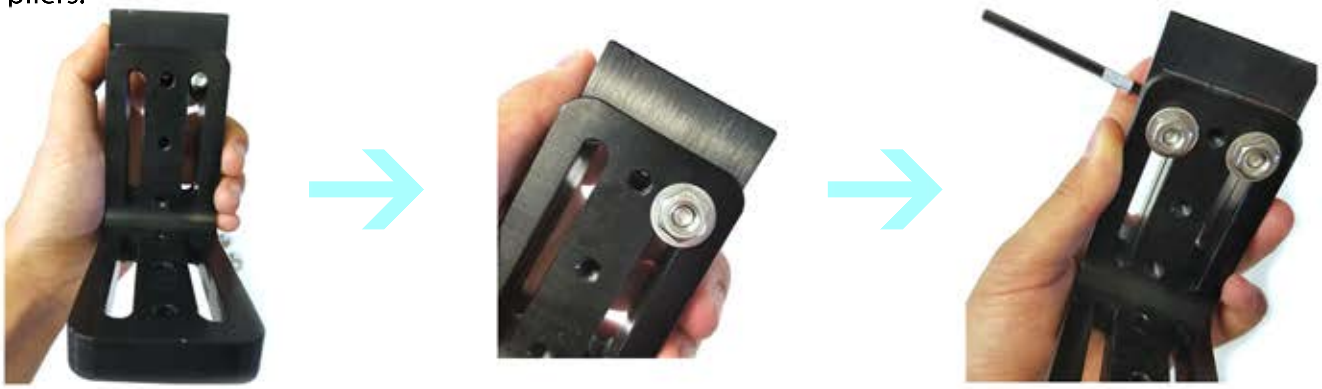

4. Screw the bracket unit into the optical breadboard with a 5/8" long, 1/4" - 20 cap screw threaded with a 1/4" flat washer. The unit should be placed to lower right handside (not centered), in order to account for offset of the headfixing shuttle. Only use one screw to hold the bracket unit to the breadboard so the unit can be rotated.

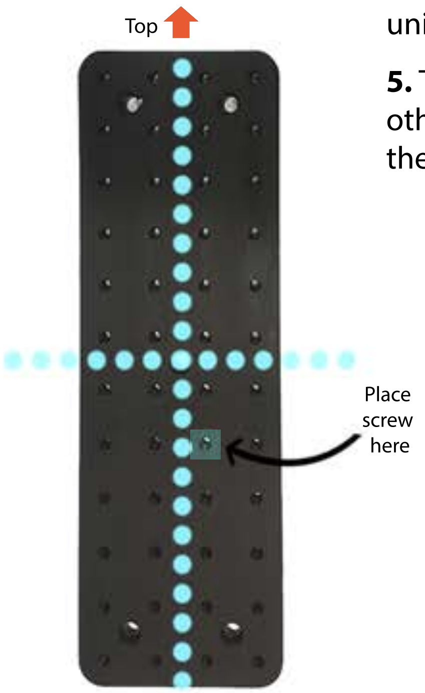

5. The kinematic base has 2 sides -- one with protruding balls and the other with wells. Screw the side with 2 wells into the screw space closest to the top of the board with a 3/4" long, 1/4" - 20 cap screw.

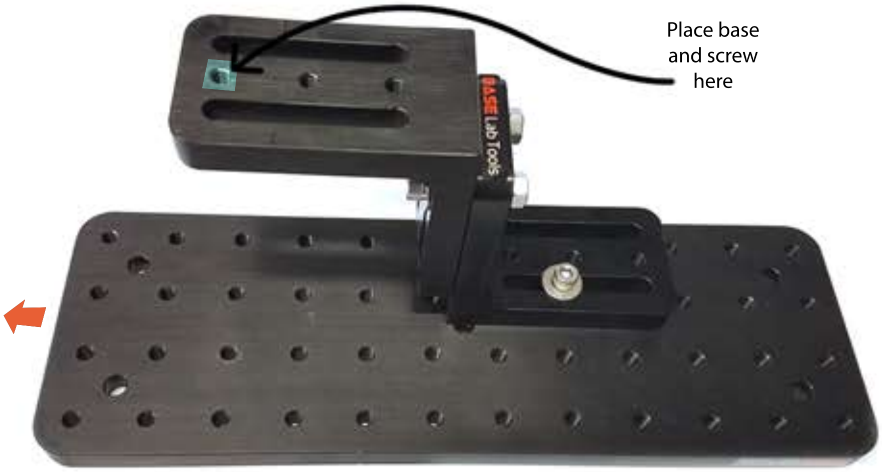

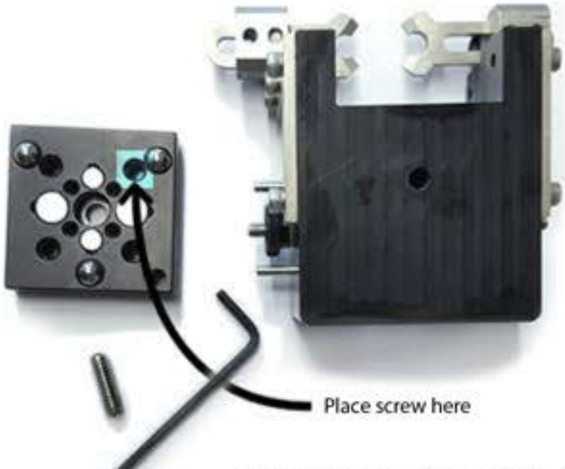

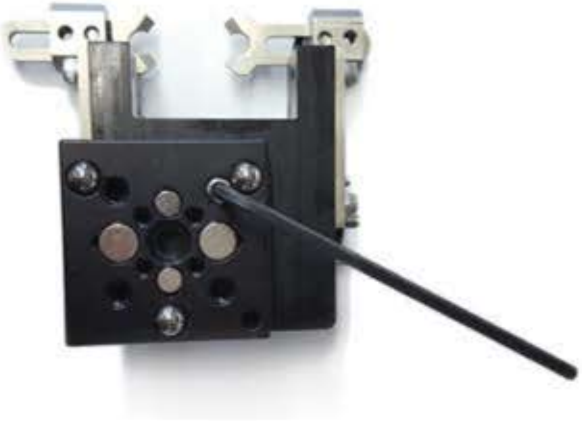

6. Screw the side with balls to the headfixing shuttle by the top right corner of the base with a set screw with the 1/8″ hex key.

7. Mark off a 50 ml plastic conical tube at 10 ml. Using a dremel or other plastic safe cutting tool, carefully drill a hole slightly larger than a 1/4″ - 20, 5/8″ long screw into the still attached cap.

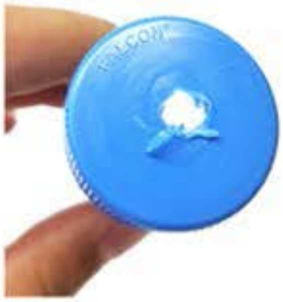

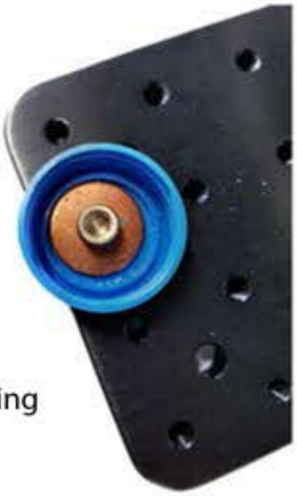

If using a dremel: Unscrew the cap from the tube, and screw upside down on the breadboard with a washer, so that the threads of the cap are pointed up. Screw the tube into place, to make a sort of stand for trimming off the pointed end of the tube at the 10 ml mark. Cut the tube by drilling into the tube, and dragging the inserted tip around the circumfrence of the tube, ensuring that the sides remain even.

8. Drill a dime sized hole in the tube, near the base of the joystick unit so the wires can be threaded through the tube. Remove wires from joystick base, and unscrew cap from the board.

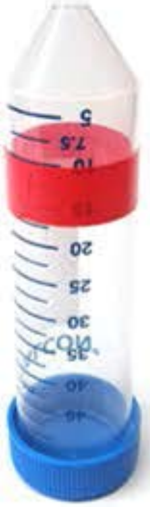

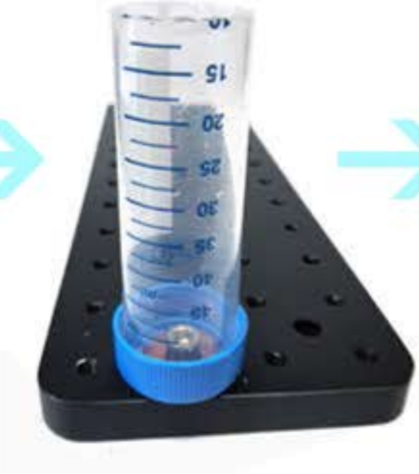

Read the manual to find out which joystick movements correspond with up/down, left/right and label before gluing into place. With the Ruffy Controls TS1 unit, "up" is in the direction of towards the wire cable input

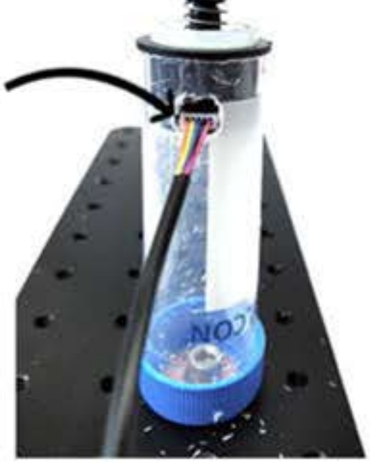

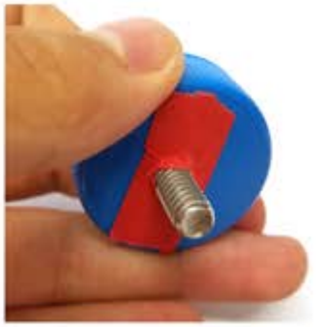

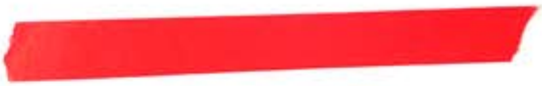

8. To make joystick bar, snip 1/16" bronze wire into 4 inch segments. Bend the wire at a 90° angle an inch from the end to form a handle.

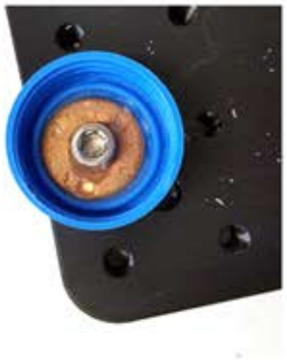

9.Tape around the bottom of the cap to prevent leaks. Place a small amount of dental acrylic between the washer and cap. Screw the cap and washer into the board to act as a clasp, and fill the bottom of the cap with dental acrylic, avoiding the screw head and threading of the cap so the rest of the tube can still be screwed in. Allow to dry completely.

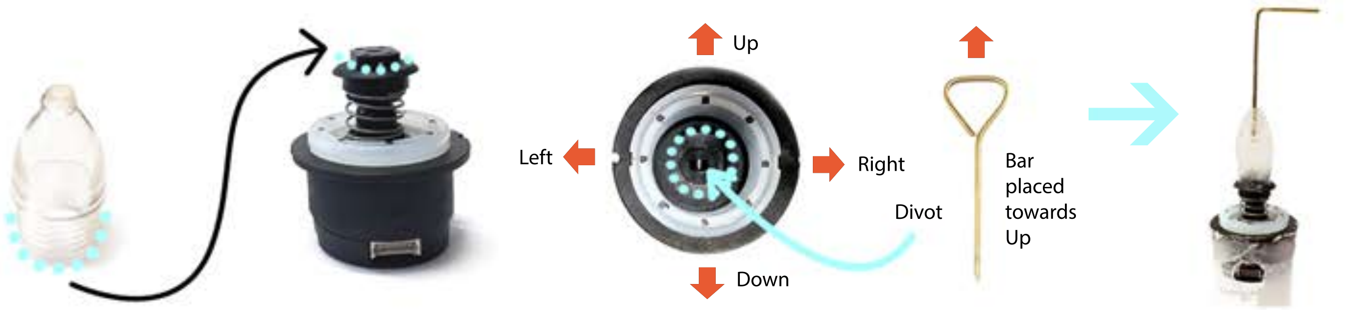

10. Remove the rubber cap from the top of the joystick unit. In order to attach the bar to the joystick unit, fill a cap that fits over joystick head with dental acrylic, and push it over the top, so that the dental acrylic settles on to the base. Push the joystick bar into the cap through the dental acrylic, and into the small central divot in the top of the joystick base. Make sure the bar and handle are straight, and that the handle is aligned with being pushed up and down. Allow to dry completely.

11. To attach the unit to the stand, smear a stiff dental acrylic mixture around the base of the unit, and the top of the stand, avoiding the hole meant for joystick connections. Push into place, while lining up the wire input and exit on the joystick and the stand. Allow to dry completely.

12. Once all pieces are dry, reassemble. Screw the base in so that the stand and joystick have "Up" pointing forward. Mark on the tube and cap what forward is, so if the stand needs to be disassembled it can be reassembled wiht ease.

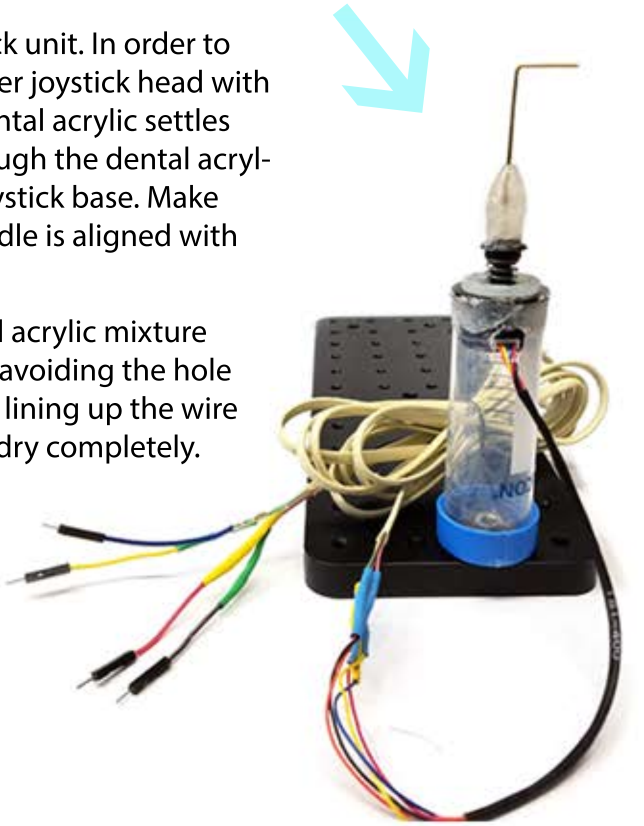

Goal: Attach joystick wires to cable wires in order to extend joystick communication to the Arduino board.

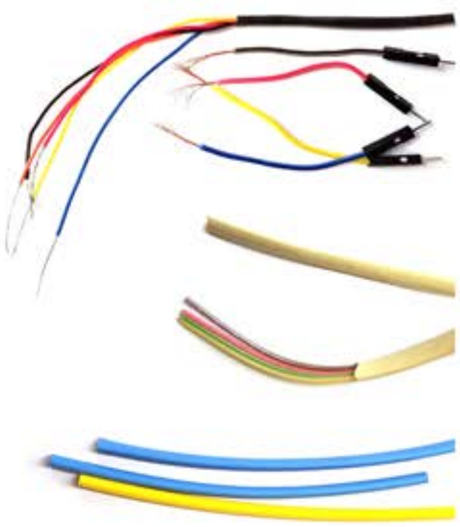

1. Strip about an inch of plastic coating from the ends of joystick wires and 4 male end jumper cables with wire strippers to expose inner metal threads. Cut away about 2 inches of plastic casing from both ends of an RJ11 cable to reveal coated wires, and strip plastic away. Take care to not break too many metal threads in the stripping process, to ensure a strong electrical connection once the soldering process is complete.

2. Check the joystick manual to see what color wire corresponds to what information sent. If certain information is uneeded (ex: not collectiong "push button" data), plan to solder that wire with the joystick "ground" wire to the RJ11 cable, because the joystick will not work if any wires are not plugged in to the Arduino board.

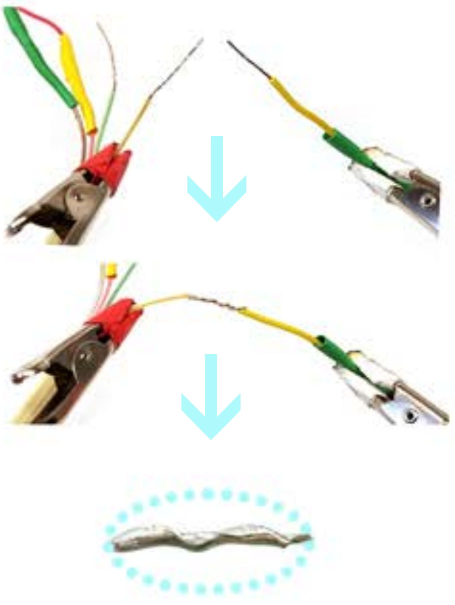

3. Heat up soldering iron, and cut shrink wrap tubing into 8, 1" pieces. With "helping hands" or another clamp system that can hold wires in proximity to each other, arrange a joystick wire across from a similarly colored wire in the cable (for clarity -- RJ11 cable colors do not have particular meanings like the joystick wires do).

To use soldering iron: Once solder wire melts when touching iron, it is hot enough to hold connections between separate wires. Dip tip of iron in soldering paste, and hold it to the exposed wire to heat. With the iron still touching the wire from behind, touch solder wire to the from right above the desired surface, so that as it the solder wire melts, it flows on to and coats the appropriate surface.

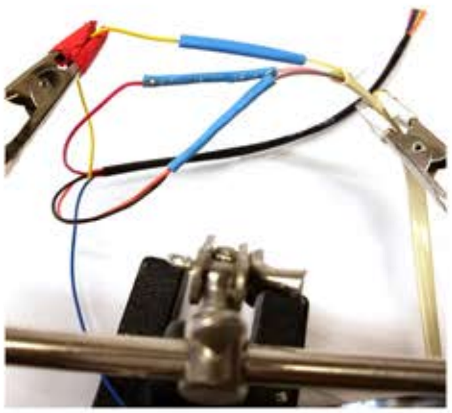

4. Thread a section of shrink wrap tubing over one of the wires. "Tin" both lengths of exposed wire by coating stripped area with a thin layer of solder. Make a mechanical conection between the 2 wires by intertwining, and secure the connection by melting a thin layer of solder over the surface, until no gaps remain. If the solder balls up, there is too much on the connection. Reheat the area until solder is liquid, and remove excess.

6. Slide the shrink wrap tubing over the junction, so that wires are not exposed. Heat the shrink tubing with the side of the unplugged soldering iron to form the plastic to the connection.

7. Repeat soldering and shrink wrap process for all other joystick wires, remembering to join uneccessary wires together with ground.

8. On the other end of the RJ11 cable, solder male ends to corresponding colors so the cable can more easily be plugged into the Arduino board.

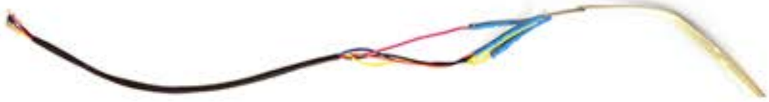

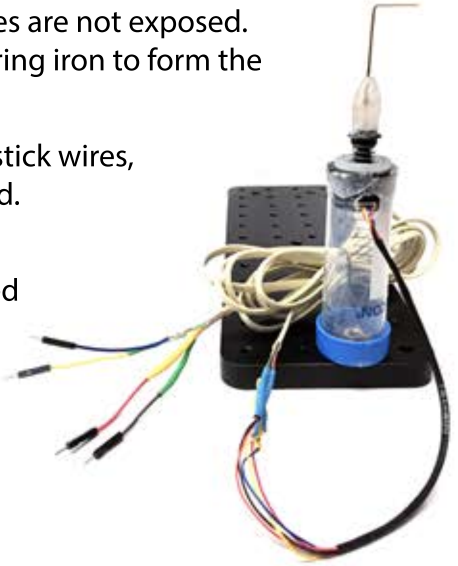

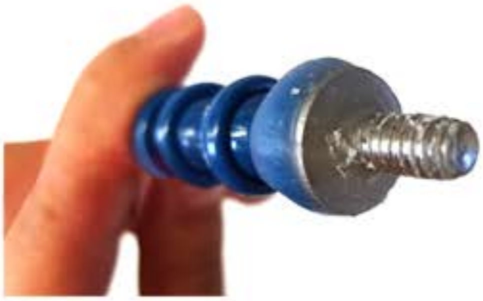

Goal: Attach screw to Loc-Line hose so that the hose can be screwed into the optical breadboard to scaffold deliver water tubing

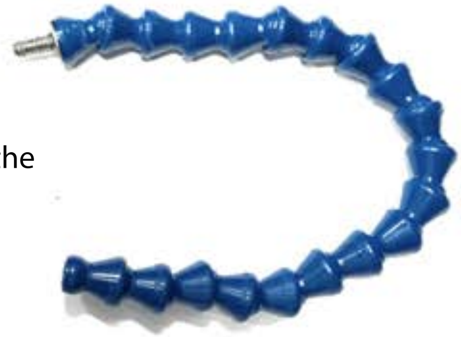

1. Mix equal parts of dental acrylic resin and liquid monomer in a disposable dish, wearing gloves. Consistency will be runny, and stiffen until completely hard (~ 10 minutes).

2. Snap together a line of approximately 15 Loc- Line pieces. Fill the base (flared end) of the last piece with dental acrylic mixture, taking care to coat sides and fill center. Embed cap screw head in the mixture, so that it is centered in the base and is surrounded by dental acrylic.

3. Push the washer over the screw, until it is aligned with the bottom of the base Loc-Line piece. Cover the washer and outside of the base with dental acrylic and mold mixture around both in order to secure the screw and washer in place.

4. When the dental acrylic has is mostly stiff, make sure screw is in a straight line with the rest of the hose and that dental acrylic is not in the threads of the screw. Hang to dry with the screw pointing down so that dental acrylic inside the base piece settles around the screw head. Allow to dry completely overnight.

5. Screw Loc-Line hose into the top left corner of the optical board. Make sure the headfixing unit and the joystick stand are aligned so the joystick bar is underneath the forks of the shuttle.

6. Affix water delivery port of choice to Loc-Line hose.

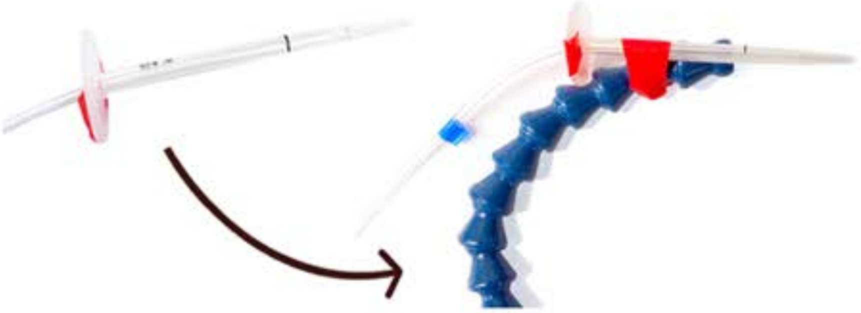

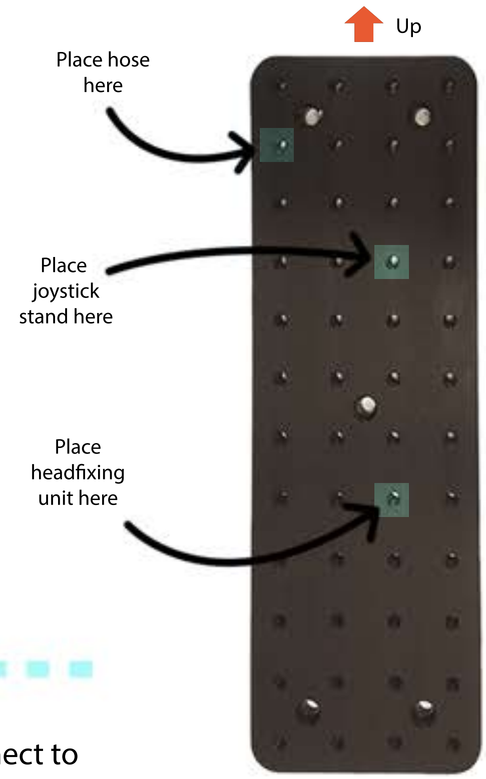

Goal: Build an Arduino controlled water delivery system and connect to joystick and headfixing unit

1. Using online instructions, create an Arduino controlled solenoid. Instructions found at:

https://www.instructables.com/id/Controlling-solenoids-with-arduino/

Look at the schematic wiring diagram to check that current is flowing through the entire circuit (ex: resistor and diodes are pointing the correct way), and that the solenoid is properly connected.

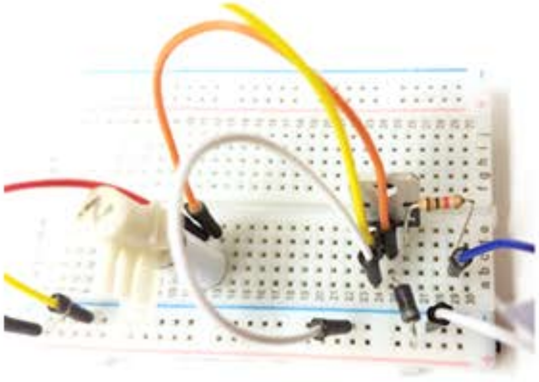

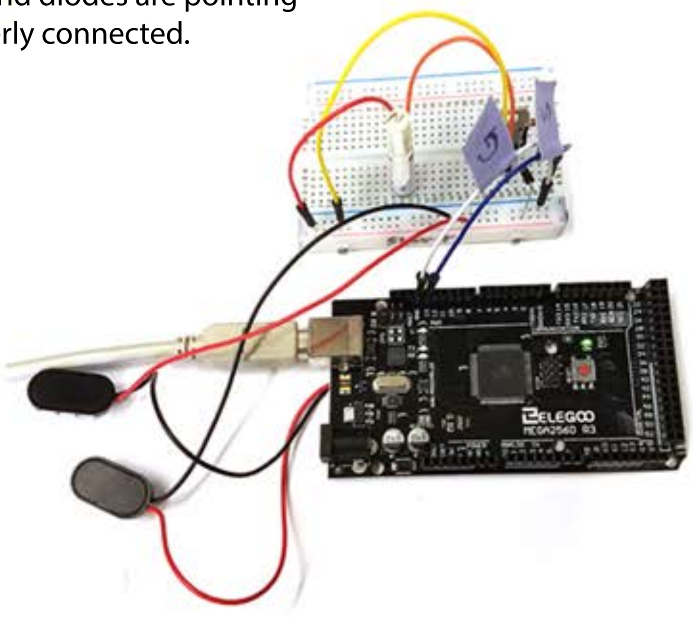

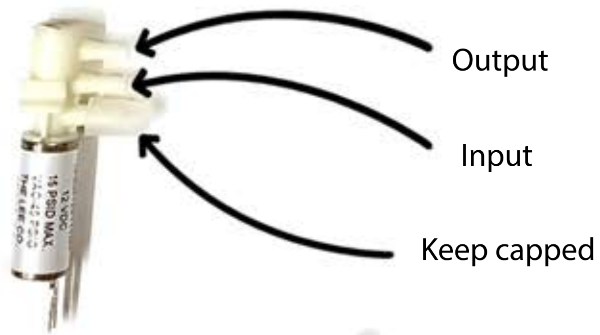

2. Once ciruit is complete, attach lick spout tubing to the output valve of the solenoid. Attach other length of Tygon tubing to the input valve. If using the 3-Port HDI solenoid, leave the third valve capped.

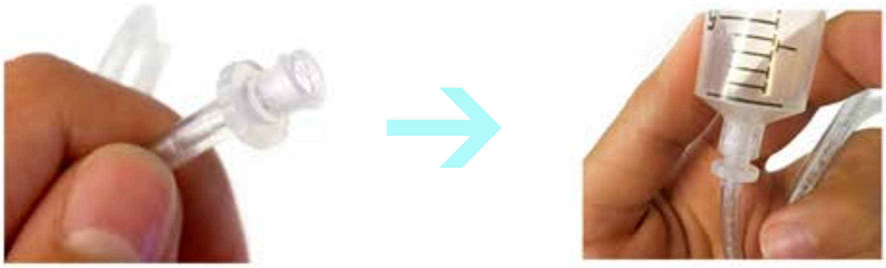

3. Push unconnected end of input valve tubing over Luer loc screw, and screw into base of Luer lock 30ml syringe.

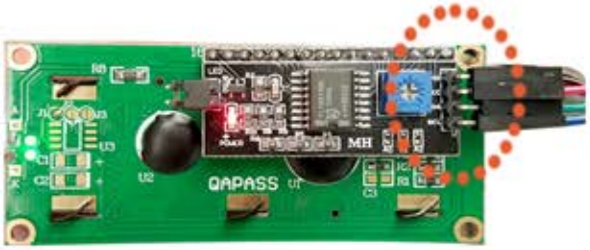

4. Connect LCD screen to the Arduino board, so that mouse progress can be tracked in real time. Using the back of the screen as a guide, connect the screen to the Arduino board through ports SCL, SDA, Ground, and 5V with four male/male jumper cables.

5. For final assembly of system, tape tube connected syringe on to a wall near where the rig will be kept. Arrange Arduino board and electrical breadboard onto optical bread-board with headfixing unit, to create a con-tained, modular training system. Secure down pieces with tape or twist ties.

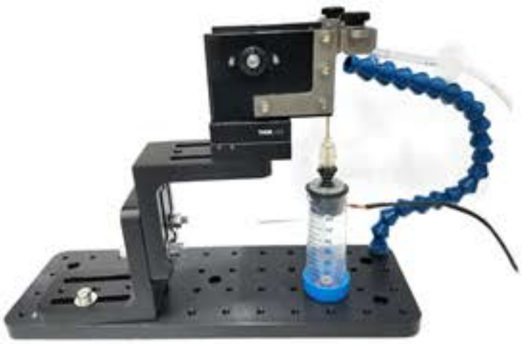

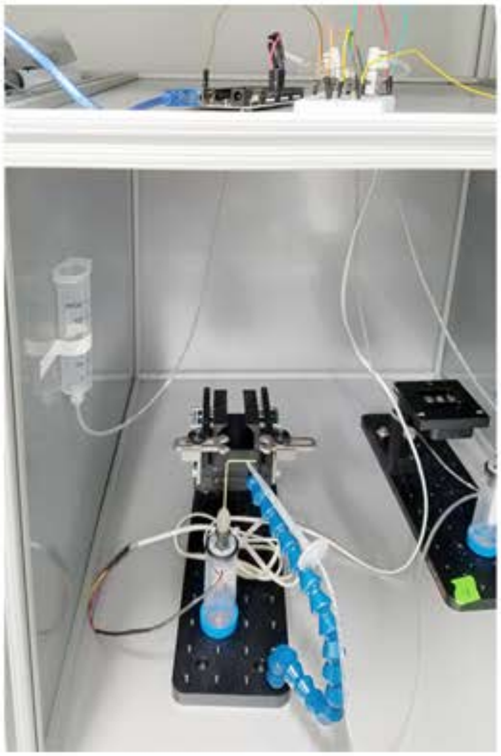

Notes on Data Collection

Because neither the Arduino IDE nor board store data, joystick information must be held in alternate programs like Processing, or on additional hardware, like micro SD cards. Through both collection methods, .csv files containing desired information can be generated and read through data processing software such as MATLAB.

Processing:
1. Set up a Processing script to write files to a certain folder path.
2. Make sure board is directly connected to computer, by way of the A/B USB cable.
3. Select the correct port in Arduino, from the pull down menu path: Tools>Port
4. To record data, run the Processing script (play button). Check that the file is actively writing to the desired folder. If it is not, stop the script, unplug/re-plug the A/B cable, and try again.

Micro SD:
1. Set up MATLAB script to pull files off of micro SD card.
2. Attach card reader to Arduino board, based on inputs (CS, SCK, MOSI, MISO, VCC, GND) on the back of the device with 6 Female/Male jumper cables.
3. When recording data, make sure micro SD card has “clicked” into place in the reader. To stop recording, take the SD card out of the reader.

Software links:
Arduino IDE – https://www.arduino.cc/en/main/software
Processing – https://processing.org/download/

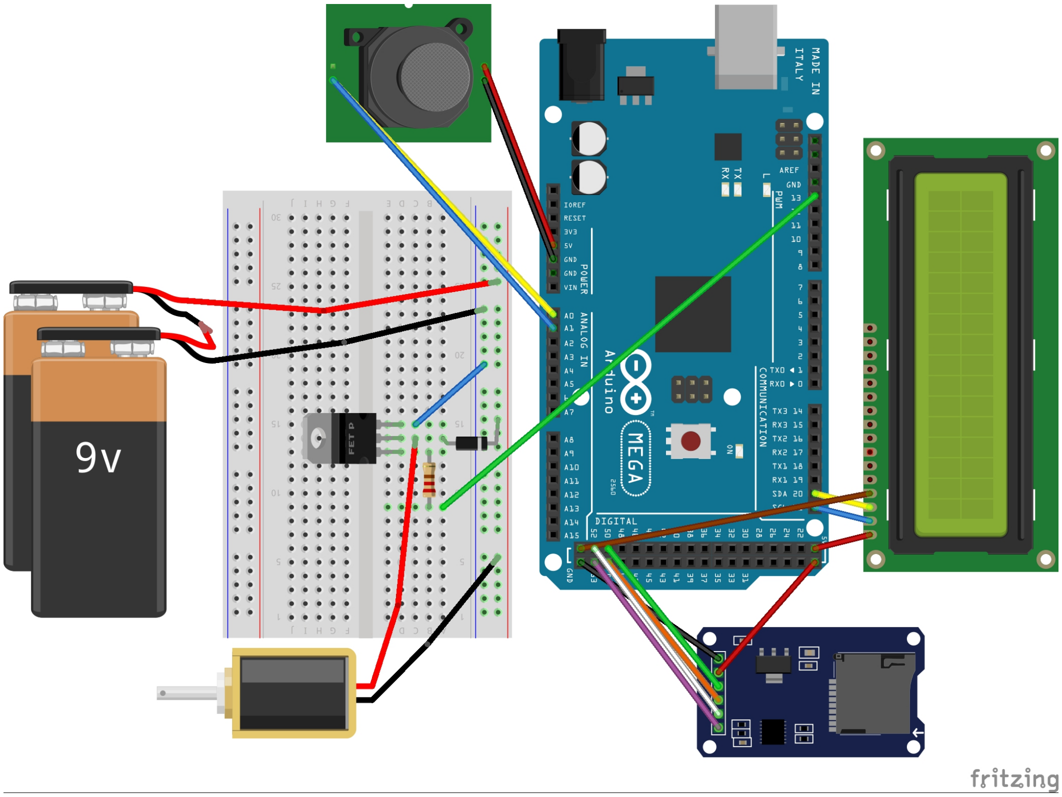

Materials Order Form

Base Lab Tools:

Item	Catalog Number	Description	Price
¼ -20 Stainless Steel Screw Kit	SKT25	Screw kit including ¼– 20 Cap Screw, Set Screws, Nuts, Washers	$49.00
Optical Breadboard	SAB0412-D	4" x 12" x 1/2" Solid Aluminum Optical Breadboard, Black Anodized	$65.00
Right Angle Mounting Bracket	ABS002	2 in Right Angle Mounting Bracket, Narrow Slotted (1)	$39.00

McMaster Carr:

Item	Catalog Number	Description	Price
0.34" ID, 0.875" OD Washer	93490A030	Bronze Washer for 5/16" Screw Size, 0.34" ID, 0.875" OD (pack of 10)	$5.70
1/16" ID, 1/8" OD Tubing	6516T11	Tygon PVC Clear Tubing 1/16" ID, 1/8" OD, 25 ft length	$5.75
1/16" Brass Wire	8859K511	Ultra-Formable 260 Brass 1 Foot Long Rod, 1/16" Diameter (pack of 3)	$2.03
Loc-Line	10095K97	Loc-Line Coolant Hose 1/4" Trade Size, Female x Male, 5 Feet Long	$28.20
Male Luer Lock 1/8" ID Hose Barb	51525K291	Plastic Quick-Turn Tube Coupling Sockets, for 1/16" Barbed Tube ID, Polypropylene (pack of 10)	$5.24

Thorlabs:

Item	Catalog Number	Description	Price
Kinematic Base	KB2X2	2" x 2" Kinematic Base, Top and Bottom Plates, 1/4"-20 Mounting	$83.39

Ruffy Controls:

Item	Catalog Number	Description	Price
Miniature 2 Axis Hall Effect Joystick	TS1-1-R-R-1-BK	TS1, Stepped Cap, Round Limiter, rear Mount, 0-5v, Black	$75.00

Items from Other Retailors:

Item	Name on Site	Website	Price
3 – Port HDI Solenoid	1303	https://sanworks.io/shop/viewproduct?productID=1303	$74.00
30 ml Syringes	30 mL Syringe Luer-Lok Tip, Box of 56	https://www.vitalitymedical.com/30-ml-syringes-without-needle.html	$25.84
50 ml Conical Tubes	14-432-22, Case of 500	https://www.fishersci.com/shop/products/falcon-50ml-conicalcentrifuge-tubes-2/p-193321	$379.25
Battery Clip Connector	9V Volt Clip on Type Battery Snap Connector Lead Wire Plastic Head, 10 Pieces	https://www.amazon.com/Battery-Connector-Plastic-Atomic-Market/dp/B00IDHZ5FM	$6.99

1K Diodes	1N5408 Rectifier Diode 3A 1000V	https://www.amazon.com/Parts-Express-1N5408-Rectifier-Diode/dp/B0009XSN02	$6.91
Electrical Breadboard	BB400 Solderless Plug-in BreadBoard, 400 tie-points, 4 power rails, 3.3 x 2.2 x 0.3in (84 x 55 x 9mm)	https://www.amazon.com/BB400-Solderless-Plug-BreadBoard-tiepoints/ dp/B0040Z1ERO	$5.90
Elegoo Board	Elegoo EL-CB-001 UNO R3 Board ATmega328P ATMEGA16U2 with USB Cable for Arduino	https://www.amazon.com/Elegoo-EL-CB-001-ATmega328PATMEGA16U2-Arduino/dp/B01EWOE0UU	$10.86
Heat Shrink Tubing, 1/8”	Polyolefin 2:1 Heat Shrink Tubing	https://www.amazon.com/Polyolefin-Heat-Shrink-Tubing-Inch/dp/B01G5RQ3KW	$16.99
Jumper Cables	40pin Male to Female, 40pin Male to Male, 40pin Female to Female Breadboard Jumper Wire Ribbon Dupont Cables Kit, 120 Pieces	https://www.amazon.com/COMeap-120pcs-Female-Breadboard-Jumper/dp/B01MU0IMFF/ref=sr_1_4?s=industrial&ie=UTF8&qid=1538699670&sr=1-4&keywords=male+male+jumper+cables	$7.99
LCD Screen	LGDehome IIC/I2C/TWI LCD 1602 16x2 Serial Interface Adapter Module Blue Backlight for Arduino UNO R3 MEGA2560 (2 pack)	https://www.amazon.com/LGDehome-Interface-Adapter-Backlight-MEGA2560/dp/B0711WLVP9	$9.59
Micro SD Card	Micro SD Card 32GB,AUAMOZ Micro SDHC Class 10 UHS-I High Speed Memory Card for Phone,Tablet and PCs - with Adapter (2 Pack)	https://www.amazon.com/gp/product/B07DGHCFSM/ref=oh_aui_search_asin_title?ie=UTF8&psc=1	$16.12
Micro SD Card Reader for Arduino	SenMod 5PCS Micro SD Card Micro SDHC Mini TF Card Adapter Reader Module for Arduino	https://www.amazon.com/gp/product/B01JYNEX56/ref=ppx_yo_dt_b_asin_title_o00__o00_s00?ie=UTF8&psc=1	$8.29
RJ11 Telephone Cable	C2G/Cables to Go 02970 RJ11 Modular Telephone Cable, Silver (7 Feet, 2.13 Meters)	https://www.amazon.com/C2GCables- Modular-Telephone-Silver/dp/B00006HSK6	$3.47
60 V Transistors	Major Brands TIP120. Transistor, Darlington, NPN, 60 Volt, 5Amp, 3-Pin, 3+ Tab, TO-220, AmpB, Rail, Pack of 15	https://www.amazon.com/Major-Brands-TIP120-Transistor-Darlington/dp/B00B888622/ref=lp_306910011_1_7?s=industrial&ie=UTF8&qid=1538700353&sr=1-7	$5.99

Approximate Cost of One Setup: $440
