# Supplementary material for: Open-Source Joystick Manipulandum for Decision-Making, Reaching, and Motor Control Studies in Mice
Source: eNeuro. 2020 Mar 24;7(2):ENEURO.0523-19.2020. doi: 10.1523/ENEURO.0523-19.2020 (PMC7131984; doi:10.1523/ENEURO.0523-19.2020)
Supplement: Extended Data 2 — Online and offline joystick code. Online and offline code can be found at the Yttri Lab GitHub (https://github.com/YttriLab/Joystick). “Arduino Code” contains sketches to run the basic center-out reaching task, the VAO task, the reaction time task, and the directional-dependent two-armed bandit task. All tasks are capable of tracking real-time joystick position and allow for experimenter defined control of task parameters. The folder also includes code to flush fluid delivery lines for cleaning. Supplied in “Processing Code” is a sketch that can be used to visualize real-time joystick position as well as task performance and variables for the basic center-out reaching task. “MATLAB Code” contains the main offline analysis code (JSAnalysis.m) for the basic center-out reaching task, which collects data pertaining to task performance as well as reach kinematics. This folder also includes accessory analysis functions and a function (SavemicroSDData.m) to save and name data collected from the joystick. Download Extended Data 2, ZIP file. [file enu-eN-OTM-0523-19-s04.zip › JS-Manual-master/JSBuildManual_Redacted.pdf]

Building a rodent joystick to monitor real-time spatiotemporal kinematics of reaching behavior

[redacted], [redacted], [redacted]

[redacted]
*Correspondence: [redacted]

SUMMARY:
We have created a step-by-step manual for inexpensive implementation and use of a rodent joystick for behavioral analysis. We have included parts list and construction instructions and links to task and analysis code for behaviors included reaching in any direction past a criterion amplitude, cued reaction time task, and a probabilistic reward 'two-arm bandit' task to two directions. These various tasks, and the method to construct and implement them, will enable greatly improved study of the neural correlates of behavior in the powerful mouse model organism. Moreover, the joystick provides a significant advantage to other reach tasks by enabling real-time behavioral monitoring and manipulations in closed-loop with ongoing behavior.

INTRODUCTION:
The study of behavioral spatiotemporal dynamics in the mouse has grown considerably in recent years (Harvey et al., 2009; Cohen et al., 2015; Guo et al., 2015; Klaus et al., 2017), opening new avenues of discovery that take advantage of the optogenetic advantages the mouse model provides. Rodent work enables high-throughput methods that accelerate discovery and capture behavioral variance, rather than ignore it (Brunton et al., 2013; Guo et al., 2015). The growth of rigorous mouse behavioral paradigms also reduces barriers to entry that may limit smaller labs and institutions. Therefore, it is advantageous for the field to establish inexpensive methods to study highly-quantifiable behavior in an awake behaving mouse (Fetsch, 2016).

Reaching is a well-studied behavior across several species (Fromm and Evarts, 1981; Churchland et al., 2012; Dean et al., 2012; Cherian et al., 2013; Yttri et al., 2013; Mathis et al., 2017). This goal-oriented behavior is a unitary, highly-quantifiable movement, whereas other tasks require several actions, like reorientation followed by locomotion across a cage (Tai et al., 2012; Lak et al., 2014;). Despite this, the behavior provides rich spatiotemporal dynamics (Bollu et al., 2018) that do not exist in other presses. Joysticks have been used for decades with both human and nonhuman primates(Thoroughman and Shadmehr, 1999; Maeda et al., 2018), and more recently with rats(Slutzky et al., 2010), and can provide a real-time readout of the X and Y trajectory. In obtaining position and speed information in real time, joysticks enable the study of ongoing correlated neural activity (Paninski, 2003; Panigrahi et al., 2015) or stimulation in closed loop triggered off a specific spatiotemporal feature of movement (Yttri and Dudman, 2016). This feature presents a significant advantage over impressive, but post-hoc, motion capture techniques (Guo et al., 2015; Mathis et al., 2018; Robie et al., 2017) – though computer vision methods are quickly advancing real-time capabilities (Ellens et al., 2016)

We describe here the pathway to build a head-fixed joystick setup – including hardware, data handling, and software. Also included are build and ordering instructions. This setup will work with any of the multiple mouse head-fixation solutions have been developed, including the RIVETS system used here (Osborne and Dudman, 2014). While already relatively inexpensive, we provide additional options to reduce cost, including using a 2-axis potentiometer joystick (~5$) in place of Hall effect joysticks (~75$). We prefer the latter, as the resistance is uniform in any direction - instead of having two axes along which there is less resilience. Additionally, the resistance of Hall effect joysticks may be decreased by cutting the spring – though care should be taken not to cut off too much (typically no more than 1.5 coils).

Specific features of the joystick system described:
- Arduino-based code for relaying multiple channels of data, including LED screen to display current task state and progress
- Rapid animal shuttle insertion, through magnetic platforms that instantly and reliably lock into a predetermined position
- Low cost, efficient build time, and small size can enable a lab to quickly and easily set up dozens of rigs in a small space.
- Task code to be run on rodents, including basic un-cued reaching, cued reaction time, and a probabilistic 'two-arm bandit' task requiring left or right reaches
- Analysis code for extracting reach performance parameters offline, including reach trajectory, amplitude, peak speed, duration, and inter-reach interval

BUILD PROTOCOL:

TASK CODE:
We have written code for several behavioral tasks common to non-human primate literature. Reaching has been utilized to study many important neural mechanisms, including the planning and generation of reaches, reaction time, and the valuation of different actions. As such, in our github repository https://github.com/JS-Manual-GitHub/JS-Manual we have provided code for:
- un-cued reaching
- cued reaction time
- probabilistic reward 'two-arm bandit' task

ANALYSIS CODE:
We have produced data analysis code to quantify the execution of reaches, including reach trajectory, amplitude, peak speed, duration, and inter-reach interval. Detection is based on threshold crossing, and then works forward and backwards from this threshold to determine exact reach initiation and termination times. In doing so, the user is able to select for only full reaches and ignore small blips due to postural adjustment, grooming, or

other non-task related behavior. For assistance with the code, we recommend contacting the lab directly in addition to visiting our github page, mentioned above.

CONCLUSION:

Studying the neural correlates of behavior requires precise, oftentimes real-time measures of those actions. Moreover, scientific apparatus often come with a hefty price, precluding high-throughput use through multiple iterations of the device (Brunton et al., 2013) or purchasing any device to begin with. We document here an inexpensive, high-performance joystick to relay the position of reaches with sub-millisecond delay. As this is an open project, we encourage feedback and will update the methods and manners of implementation as our process matures.

ACKNOWLEDGEMENTS:

[redacted], [redacted], and especially [redacted] and his group at [redacted]

REFERENCES:

Bollu, T., Whitehead, S.C., Prasad, N., Walker, J.R., Shyamkumar, N., Subramaniam, R., Kardon, B.M., Cohen, I., Goldberg, J.H., 2018. Cortical control of kinematic primitives in mice performing a hold-still-center-out reach task. bioRxiv 304907. https://doi.org/10.1101/304907

Brunton, B.W., Botvinick, M.M., Brody, C.D., 2013. Rats and humans can optimally accumulate evidence for decision-making. Science (80-.). https://doi.org/10.1126/science.1233912

Cherian, A., Fernandes, H.L., Miller, L.E., 2013. Primary motor cortical discharge during force field adaptation reflects muscle-like dynamics. J. Neurophysiol. 110, 768–783. https://doi.org/10.1152/jn.00109.2012

Churchland, M.M., Cunningham, J.P., Kaufman, M.T., Foster, J.D., Nuyujukian, P., Ryu, S.I., Shenoy, K. V., Shenoy, K. V., 2012. Neural population dynamics during reaching. Nature. https://doi.org/10.1038/nature11129

Cohen, J.Y., Amoroso, M.W., Uchida, N., 2015. Serotonergic neurons signal reward and punishment on multiple timescales. Elife 1–25. https://doi.org/10.7554/eLife.06346

Dean, H.L., Hagan, M.A., Pesaran, B., 2012. Only Coherent Spiking in Posterior Parietal Cortex Coordinates Looking and Reaching. Neuron 73, 829–841. https://doi.org/10.1016/j.neuron.2011.12.035

Ellens, D.J., Gaidica, M., Toader, A., Peng, S., Shue, S., John, T., Bova, A., Leventhal, D.K., 2016. An automated rat single pellet reaching system with high-speed video capture. J. Neurosci. Methods. https://doi.org/10.1016/j.jneumeth.2016.07.009

Fetsch, C.R., 2016. The importance of task design and behavioral control for understanding the neural basis of cognitive functions. Curr. Opin. Neurobiol. 37, 16–22. https://doi.org/10.1016/j.conb.2015.12.002

Fromm, C., Evarts, E. V, 1981. Relation of size and activity of motor cortex pyramidal tract neurons during skilled movements in the monkey. J. Neurosci. 1, 453–460.

Guo, J.-Z., Graves, A.R., Guo, W.W., Zheng, J., Lee, A., Rodriguez-Gonzalez, J., Li, N., Macklin, J.J., Phillips, J.W., Mensh, B.D., Branson, K., Hantman, A.W., 2015. Cortex commands the performance of skilled movement. Elife 4, e10774.

https://doi.org/10.7554/eLife.10774
Harvey, C.D., Collman, F., Dombeck, D.A., Tank, D.W., 2009. Intracellular dynamics of hippocampal place cells during virtual navigation. Nature. https://doi.org/10.1038/nature08499
Klaus, A., Martins, G.J., Paixao, V.B., Zhou, P., Paninski, L., Costa, R.M., 2017. The Spatiotemporal Organization of the Striatum Encodes Action Space. Neuron 95, 1171–1180.e7. https://doi.org/10.1016/j.neuron.2017.08.015
Lak, A., Costa, G.M., Romberg, E., Koulakov, A.A., Mainen, Z.F., Kepecs, A., 2014. Orbitofrontal cortex is required for optimal waiting based on decision confidence. Neuron. https://doi.org/10.1016/j.neuron.2014.08.039
Maeda, R.S., Cluff, T., Gribble, P.L., Pruszynski, J.A., 2018. Feedforward and feedback control share an internal model of the arm's dynamics. J. Neurosci. https://doi.org/10.1523/JNEUROSCI.1709-18.2018
Mathis, A., Mamidanna, P., Cury, K.M., Abe, T., Murthy, V.N., Mathis, M.W., Bethge, M., 2018. DeepLabCut: markerless pose estimation of user-defined body parts with deep learning. Nat. Neurosci. https://doi.org/10.1038/s41593-018-0209-y
Mathis, M.W., Mathis, A., Uchida, N., 2017. Somatosensory Cortex Plays an Essential Role in Forelimb Motor Adaptation in Mice. Neuron 93, 1493–1503.e6. https://doi.org/10.1016/j.neuron.2017.02.049
Osborne, J.E., Dudman, J.T., 2014. RIVETS: A mechanical system for in vivo and in vitro electrophysiology and imaging. PLoS One. https://doi.org/10.1371/journal.pone.0089007
Panigrahi, B., Martin, K.A., Li, Y., Graves, A.R., Vollmer, A., Olson, L., Mensh, B.D., Karpova, A.Y., Dudman, J.T., 2015. Dopamine Is Required for the Neural Representation and Control of Movement Vigor. Cell 162, 1418–1430. https://doi.org/10.1016/j.cell.2015.08.014
Paninski, L., 2003. Spatiotemporal Tuning of Motor Cortical Neurons for Hand Position and Velocity. J. Neurophysiol. 91, 515–532. https://doi.org/10.1152/jn.00587.2002
Robie, A.A., Seagraves, K.M., Egnor, S.E.R., Branson, K., 2017. Machine vision methods for analyzing social interactions. J. Exp. Biol. https://doi.org/10.1242/jeb.142281
Slutzky, M.W., Jordan, L.R., Bauman, M.J., Miller, L.E., 2010. A new rodent behavioral paradigm for studying forelimb movement. J. Neurosci. Methods 192, 228–232. https://doi.org/10.1016/j.jneumeth.2010.07.040
Tai, L.H., Lee, A.M., Benavidez, N., Bonci, A., Wilbrecht, L., 2012. Transient stimulation of distinct subpopulations of striatal neurons mimics changes in action value. Nat. Neurosci. 15, 1281–1289. https://doi.org/10.1038/nn.3188
Thoroughman, K.A., Shadmehr, R., 1999. Electromyographic correlates of learning an internal model of reaching movements. J. Neurosci. 19, 8573–8588.
Yttri, E.A., Dudman, J.T., 2016. Opponent and bidirectional control of movement velocity in the basal ganglia. Nature 533, 402–406. https://doi.org/10.1038/nature17639
Yttri, E.A., Liu, Y., Snyder, L.H., Goldberg, M.E., 2013. Lesions of cortical area LIP affect reach onset only when the reach is accompanied by a saccade, revealing an active eye–hand coordination circuit. PNAS 110, 2371–2376. https://doi.org/10.1073/pnas.1220508110

Table of Contents

Material List……………………………………………………………………… 1- 2

Assembly

Headfixing Unit.…………………………………………………………….3 - 4

Joystick Stand………………………………………………………………… 5 - 6

Data Collection……………………………………………………………… 7 - 8, 10

Notes………………………………………………………………………. 11

Water Delivery…………………………………………………………………… 8 – 10

Materials Order Form……………………………………………………………..12 -13

Materials List: Headfixing Unit and Joystick Module

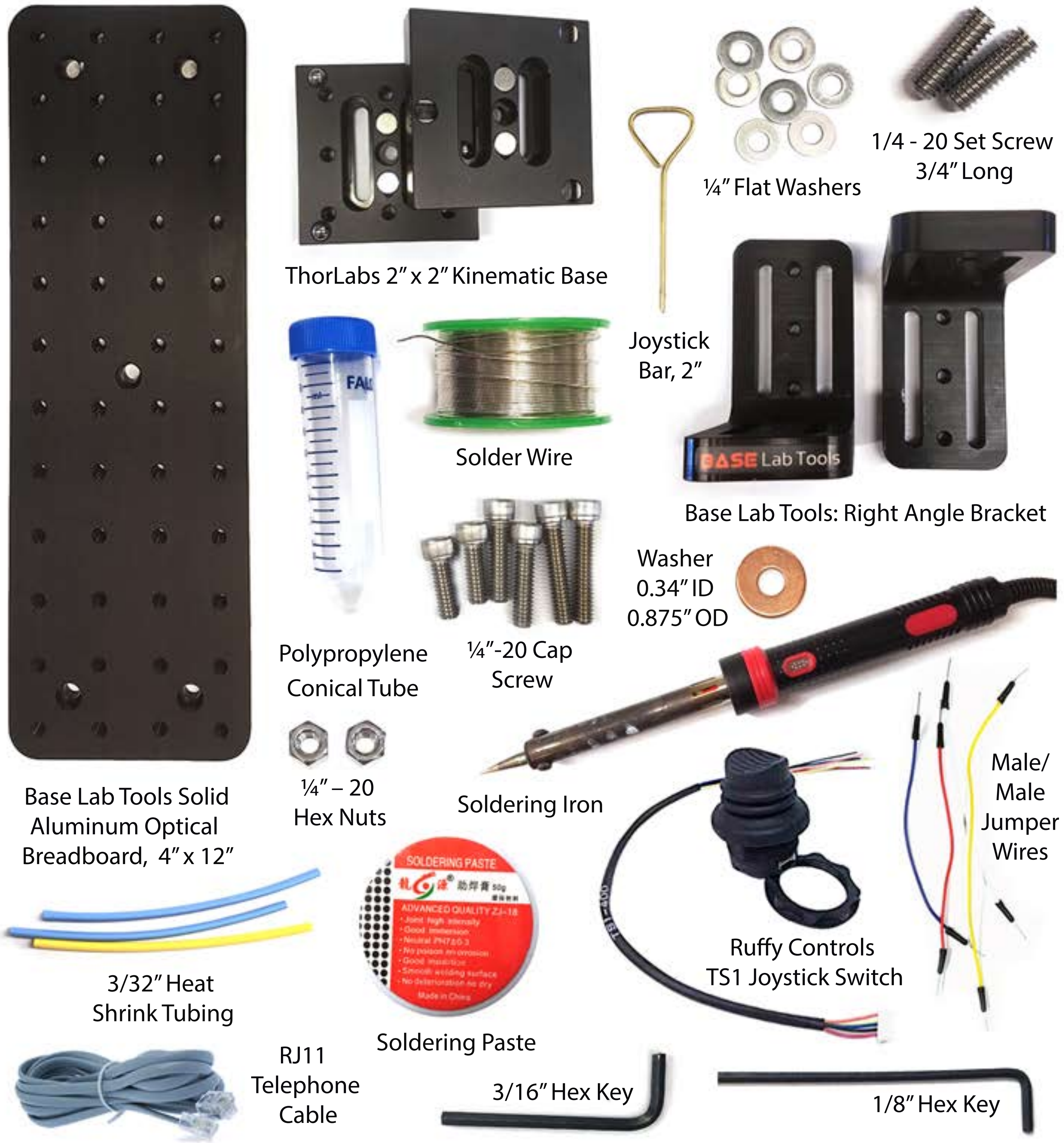

• 1, Polypropylene Conical Tube • 1, Base Lab Tools: Solid Aluminum Optical Breadboard, 4″ x 12″ • 2, Base Lab Tools: Right Angle Bracket, Narrow Slotted (ABS002) • 2, ¼″-20 Cap Screw, 1″ Long • 2, ¼″-20 Cap Screw, 1¼″ Long • 2, ¼″-20 Cap Screw, ¾″ Long • 7, ¼″ Flat Washers • 2, ¼″ – 20 Hex Nuts • 2, 1-4 -20 Set Screws, 3/4“ Long • 1, Ruffy Controls TS1 series joystick unit • 4″, 1/16“ Bronze Wire (for joystick bar) • Tape • 3/16″ Hex Key • 1/8″ Hex Key • Dental Acrylic, Resin and Liquid Monomer • Soldering Paste • Solder

Materials List: Data Collection and Water Delivery

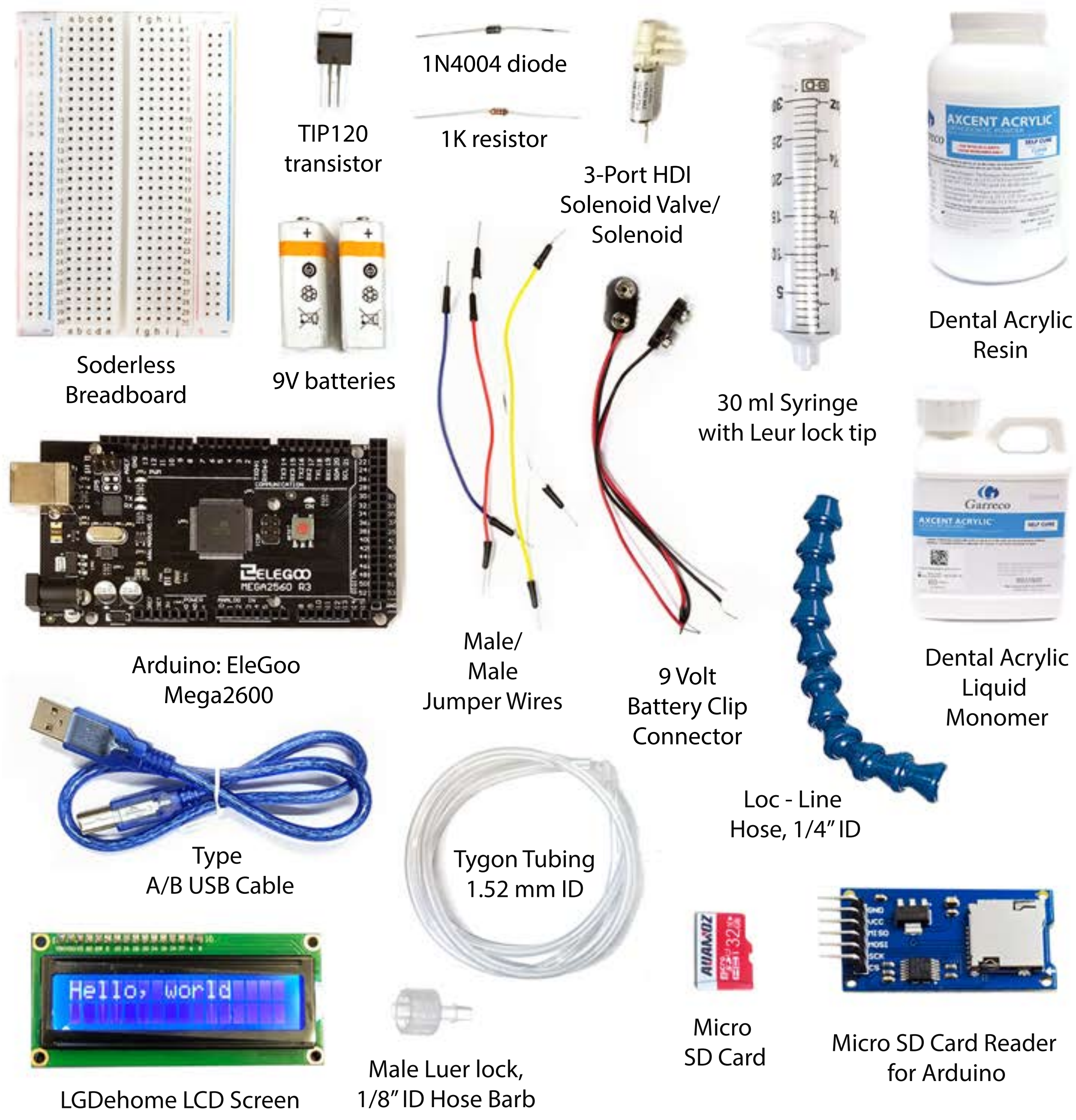

• 1, The Lee Co. 3-Port HDI Solenoid Valve (LHDA1231115H) • 2 lengths [to fit rig box], Tygon Tubing 1.52 mm ID • 1 Arduino board (EleGoo Mega2600) • 1,Type A/B USB Cable • 1, Soderless Breadboard • 2 Battery Clip Connectors • 1, 1K Resistor • 1, TIP120 transistor (or TIP102) • 1, 1N4004 diode (or 1N4001) • 2, 9V batteries • 8, Male/Male Jumper Wires • 6, Female/Male Jumper Wires • Loc - Line Modular Hose , 1/4” ID (apprx 15) • 1, LGDehome 16x2 Serial Interface Adapter for Arduino MEGA2560 • 1, Male Luer Lock, 1/8" ID Hose Barb • 1, Micro SD • 1, Micro SD Reader for Arduino • 30ml Syringe Sterile with Luer Lock Tip • Tape • Superglue • Dental Acrylic, Resin and Liquid Monomer

Goal: Build a headfixing device suitable for mice in electrophysiology experiments

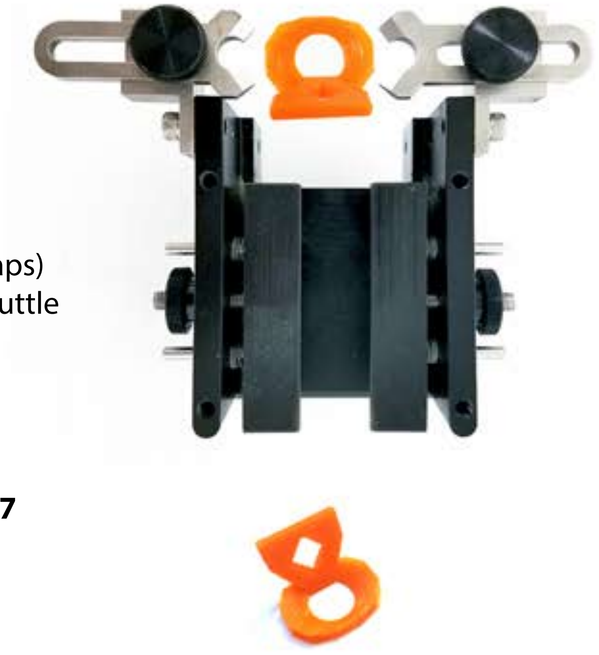

1. Using AutoCAD files and instructions found on:

http://dudmanlab.org/html/rivets_designs.html

manufacture RIVETs stystem parts (forks, desired head caps) and shuttle components. Assemble into an adjustable shuttle with headfixing capabilities.

2. Based on the Methods section of:

Osborne & Dudman (2014) PLoS One 9(2): e89007

permanently attach head caps to experimental mice.

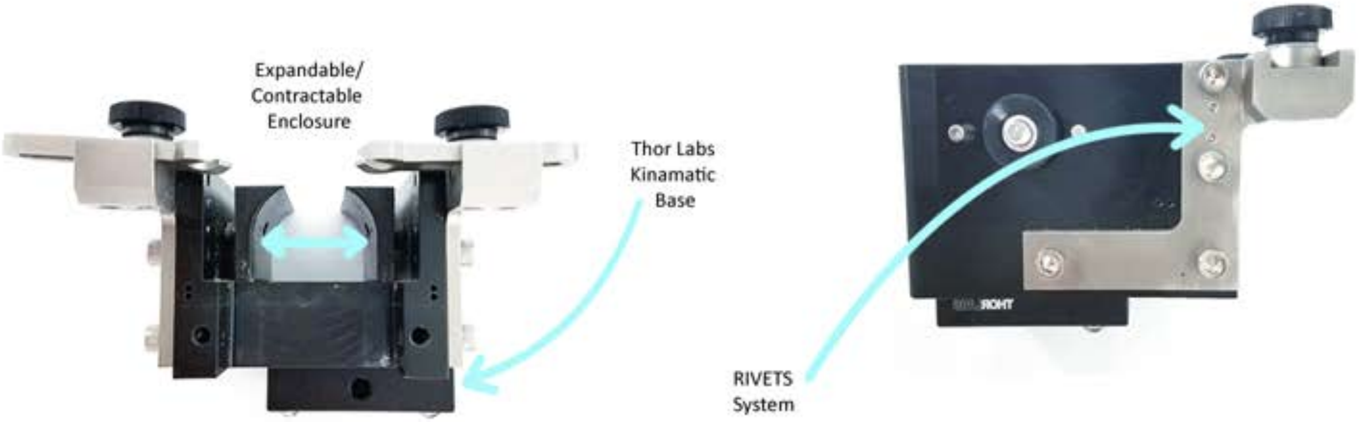

Goal: Build base and stand for headfixing shuttle.

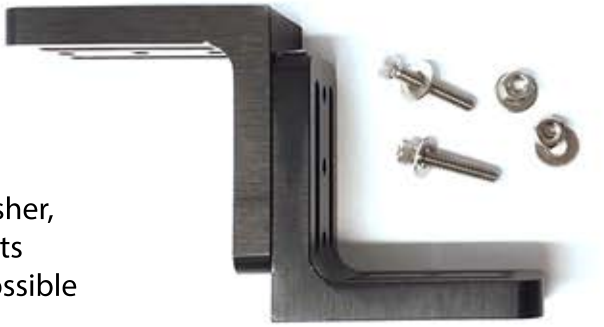

Steps:

1. Hold 2 right angle brackets in a "Z" shape, so that the slots align. Thread a 1 1/4" long, 1/4" - 20 cap screw with a 1/4" flat washer, and push through both bracket slots.

2. Cap the emerging end of the screw with a 1/4" flat washer, and then a 1/4" - 20 hex nut in order to hold the 2 brackets together. Join the pieces at the lowest extension level possible so adjustments can be made in later steps.

3. Repeat steps 1 and 2 with the other slot for security. In order to fully tighten hex nuts onto the screws hold the cap of the scew in place with a 3/16" hex key, and tighten the nut with a wrench or pliers.

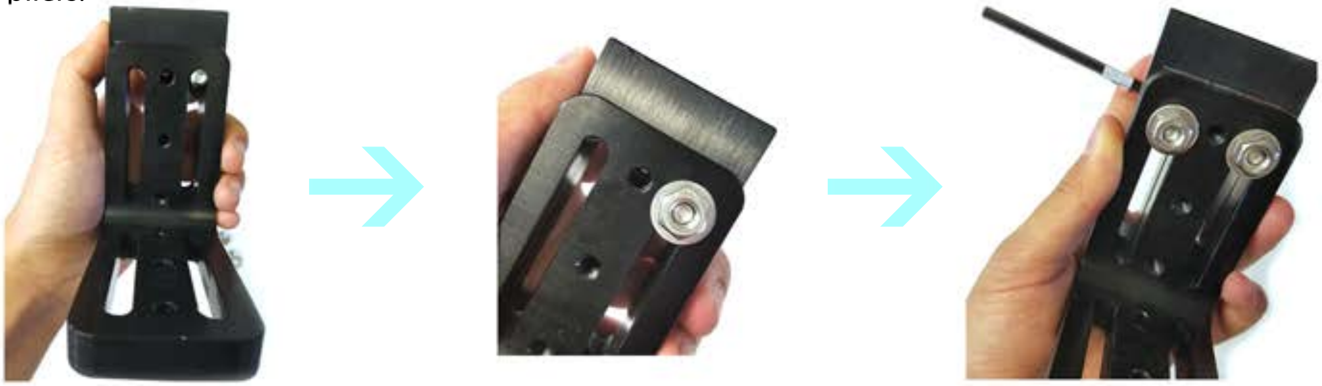

4. Screw the bracket unit into the optical breadboard with a 5/8" long, 1/4" - 20 cap screw threaded with a 1/4" flat washer. The unit should be placed to lower right handside (not centered), in order to account for offset of the headfixing shuttle. Only use one screw to hold the bracket unit to the breadboard so the unit can be rotated.

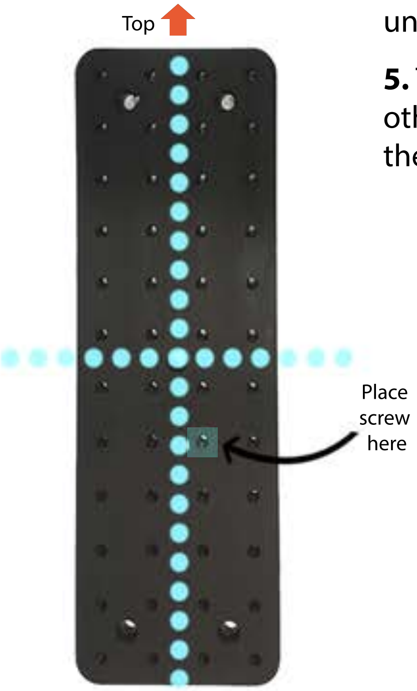

5. The kinematic base has 2 sides -- one with protruding balls and the other with wells. Screw the side with 2 wells into the screw space closest to the top of the board with a 3/4" long, 1/4" - 20 cap screw.

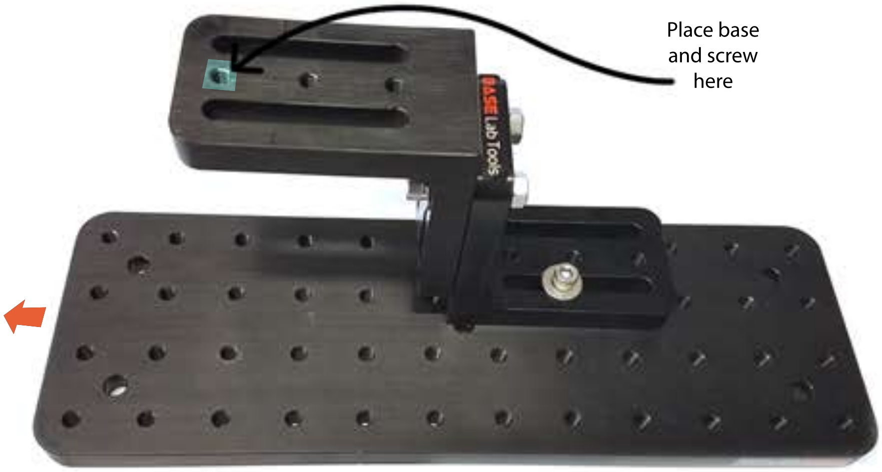

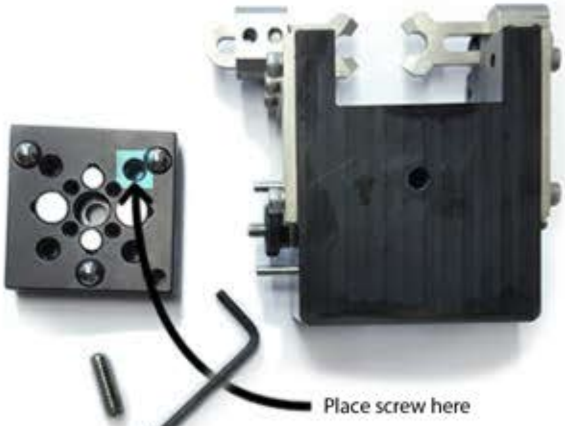

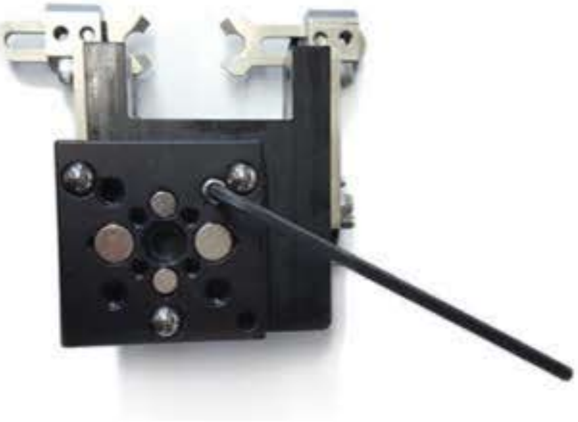

6. Screw the side with balls to the headfixing shuttle by the top right corner of the base with a set screw with the 1/8" hex key.

7. Mark off a 50 ml plastic conical tube at 10 ml. Using a dremel or other plastic safe cutting tool, carefully drill a hole slightly larger than a 1/4" - 20, 5/8" long screw into the still attached cap.

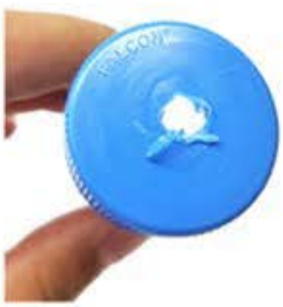

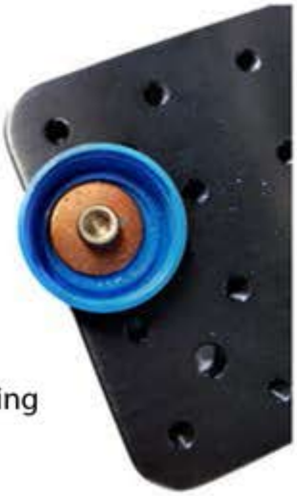

If using a dremel: Unscrew the cap from the tube, and screw upside down on the breadboard with a washer, so that the threads of the cap are pointed up. Screw the tube into place, to make a sort of stand for trimming off the pointed end of the tube at the 10 ml mark. Cut the tube by drilling into the tube, and dragging the inserted tip around the circumfrence of the tube, ensuring that the sides remain even.

8. Drill a dime sized hole in the tube, near the base of the joystick unit so the wires can be threaded through the tube. Remove wires from joystick base, and unscrew cap from the board.

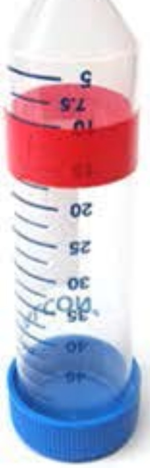

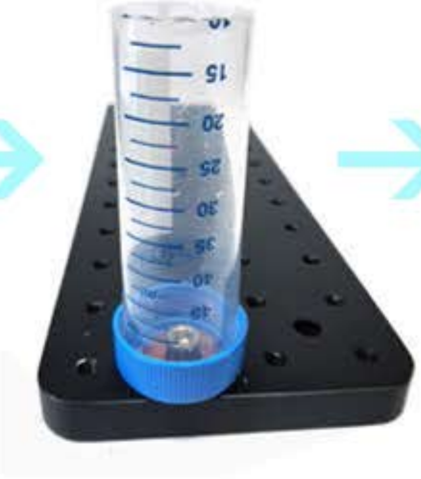

Read the manual to find out which joystick movements correspond with up/down, left/right and label before gluing into place. With the Ruffy Controls TS1 unit, "up" is in the direction of towards the wire cable input

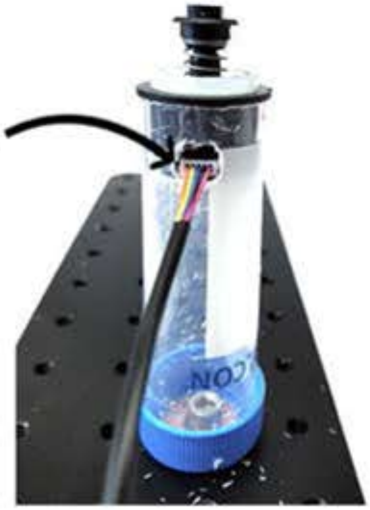

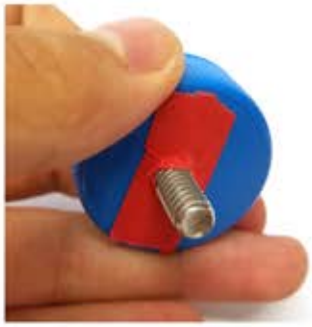

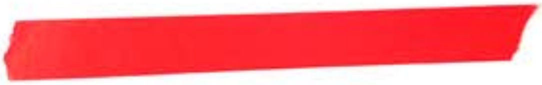

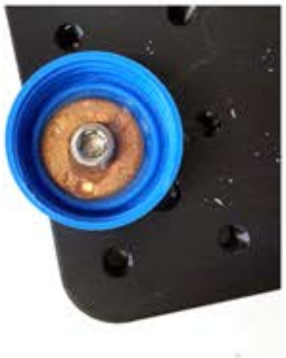

8. To make joystick bar, snip 1/16" bronze wire into 4 inch segments. Bend the wire at a 90° angle an inch from the end to form a handle.

9.Tape around the bottom of the cap to prevent leaks. Place a small amount of dental acrylic between the washer and cap. Screw the cap and washer into the board to act as a clasp, and fill the bottom of the cap with dental acrylic, avoiding the screw head and threading of the cap so the rest of the tube can still be screwed in. Allow to dry completely.

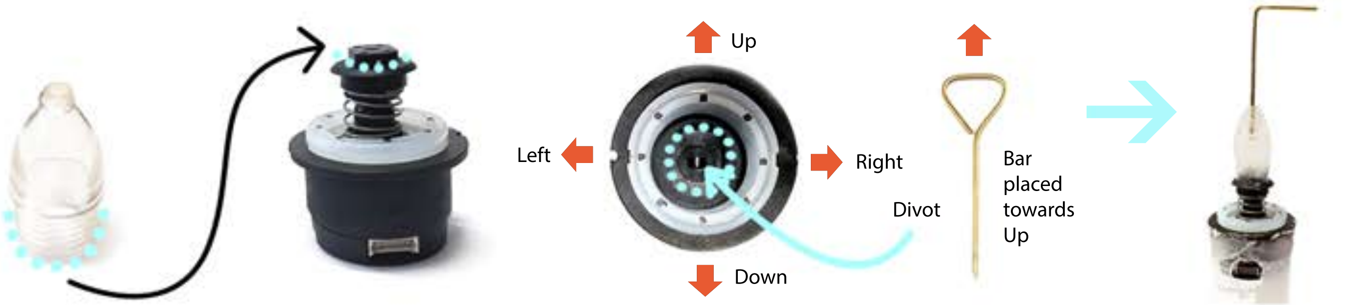

10. Remove the rubber cap from the top of the joystick unit. In order to attach the bar to the joystick unit, fill a cap that fits over joystick head with dental acrylic, and push it over the top, so that the dental acrylic settles on to the base. Push the joystick bar into the cap through the dental acrylic, and into the small central divot in the top of the joystick base. Make sure the bar and handle are straight, and that the handle is aligned with being pushed up and down. Allow to dry completely.

11. To attach the unit to the stand, smear a stiff dental acrylic mixture around the base of the unit, and the top of the stand, avoiding the hole meant for joystick connections. Push into place, while lining up the wire input and exit on the joystick and the stand. Allow to dry completely.

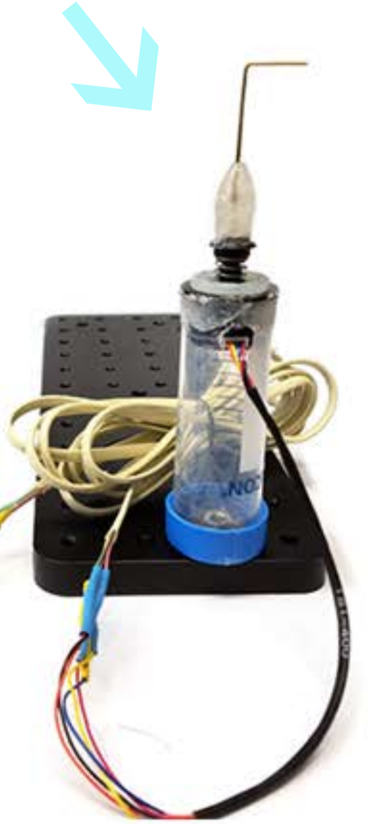

12. Once all pieces are dry, reassemble. Screw the base in so that the stand and joystick have "Up" pointing forward. Mark on the tube and cap what forward is, so if the stand needs to be disassembled it can be reassembled wiht ease.

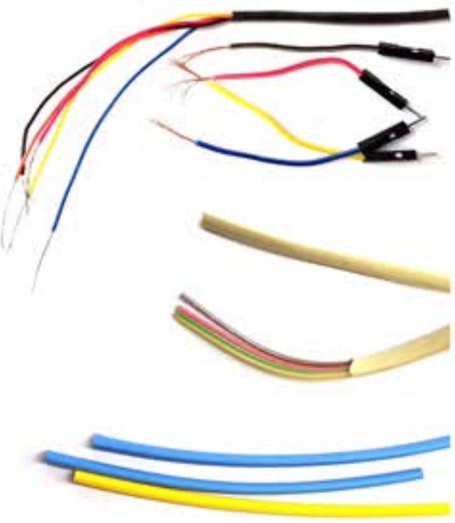

Goal: Attach joystick wires to cable wires in order to extend joystick communication to the Arduino board.

1. Strip about an inch of plastic coating from the ends of joystick wires and 4 male end jumper cables with wire strippers to expose inner metal threads. Cut away about 2 inches of plastic casing from both ends of an RJ11 cable to reveal coated wires, and strip plastic away. Take care to not break too many metal threads in the stripping process, to ensure a strong electrical connection once the soldering process is complete.

2. Check the joystick manual to see what color wire corresponds to what information sent. If certain information is uneeded (ex: not collectiong "push button" data), plan to solder that wire with the joystick "ground" wire to the RJ11 cable, because the joystick will not work if any wires are not plugged in to the Arduino board.

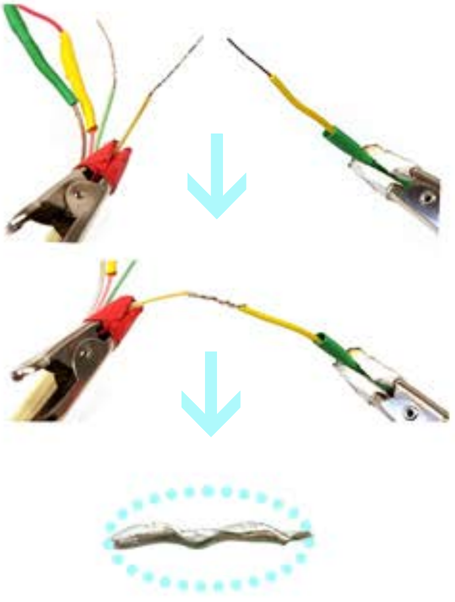

3. Heat up soldering iron, and cut shrink wrap tubing into 8, 1" pieces. With "helping hands" or another clamp system that can hold wires in proximity to each other, arrange a joystick wire across from a similarly colored wire in the cable (for clarity -- RJ11 cable colors do not have particular meanings like the joystick wires do).

To use soldering iron: Once solder wire melts when touching iron, it is hot enough to hold connections between separate wires. Dip tip of iron in soldering paste, and hold it to the exposed wire to heat. With the iron still touching the wire from behind, touch solder wire to the from right above the desired surface, so that as it the solder wire melts, it flows on to and coats the appropriate surface.

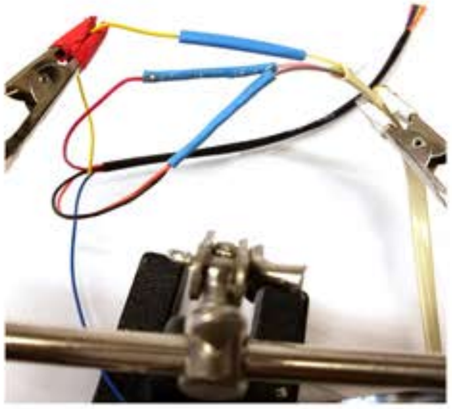

4. Thread a section of shrink wrap tubing over one of the wires. "Tin" both lengths of exposed wire by coating stripped area with a thin layer of solder. Make a mechanical conection between the 2 wires by intertwining, and secure the connection by melting a thin layer of solder over the surface, until no gaps remain. If the solder balls up, there is too much on the connection. Reheat the area until solder is liquid, and remove excess.

6. Slide the shrink wrap tubing over the junction, so that wires are not exposed. Heat the shrink tubing with the side of the unplugged soldering iron to form the plastic to the connection.

7. Repeat soldering and shrink wrap process for all other joystick wires, remembering to join uneccessary wires together with ground.

8. On the other end of the RJ11 cable, solder male ends to corresponding colors so the cable can more easily be plugged into the Arduino board.

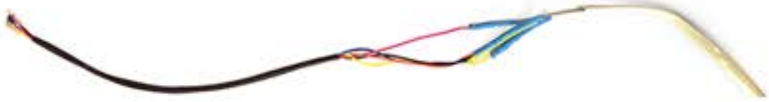

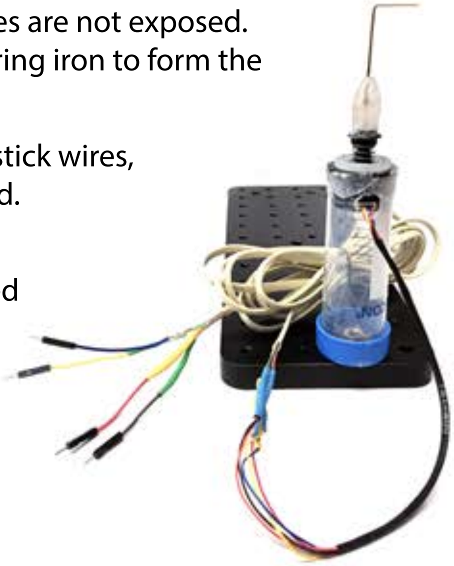

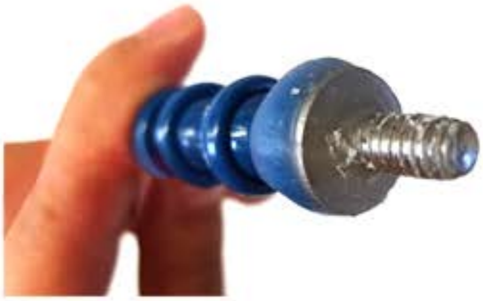

Goal: Attach screw to Loc-Line hose so that the hose can be screwed into the optical breadboard to scaffold deliver water tubing

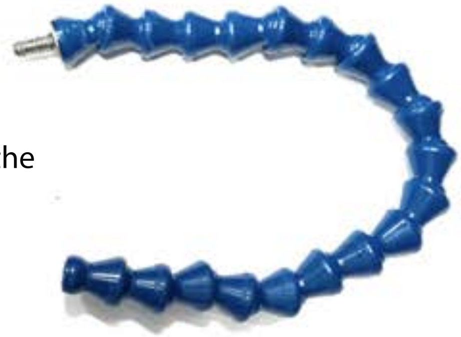

1. Mix equal parts of dental acrylic resin and liquid monomer in a disposable dish, wearing gloves. Consistency will be runny, and stiffen until completely hard (~ 10 minutes).

2. Snap together a line of approximately 15 Loc- Line pieces. Fill the base (flared end) of the last piece with dental acrylic mixture, taking care to coat sides and fill center. Embed cap screw head in the mixture, so that it is centered in the base and is surrounded by dental acrylic.

3. Push the washer over the screw, until it is aligned with the bottom of the base Loc-Line piece. Cover the washer and outside of the base with dental acrylic and mold mixture around both in order to secure the screw and washer in place.

4. When the dental acrylic has is mostly stiff, make sure screw is in a straight line with the rest of the hose and that dental acrylic is not in the threads of the screw. Hang to dry with the screw pointing down so that dental acrylic inside the base piece settles around the screw head. Allow to dry completely overnight.

5. Screw Loc-Line hose into the top left corner of the optical board. Make sure the headfixing unit and the joystick stand are aligned so the joystick bar is underneath the forks of the shuttle.

6. Affix water delivery port of choice to Loc-Line hose.

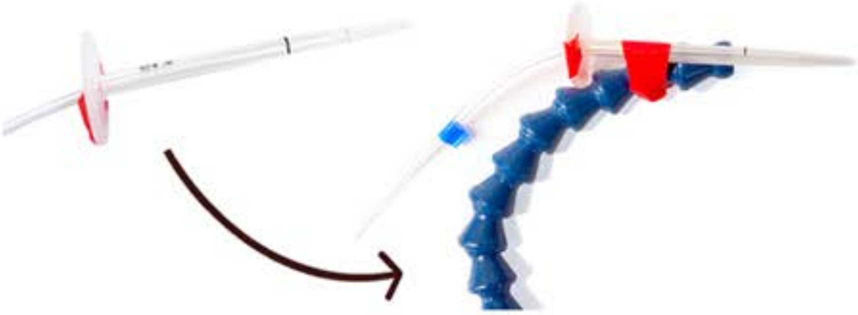

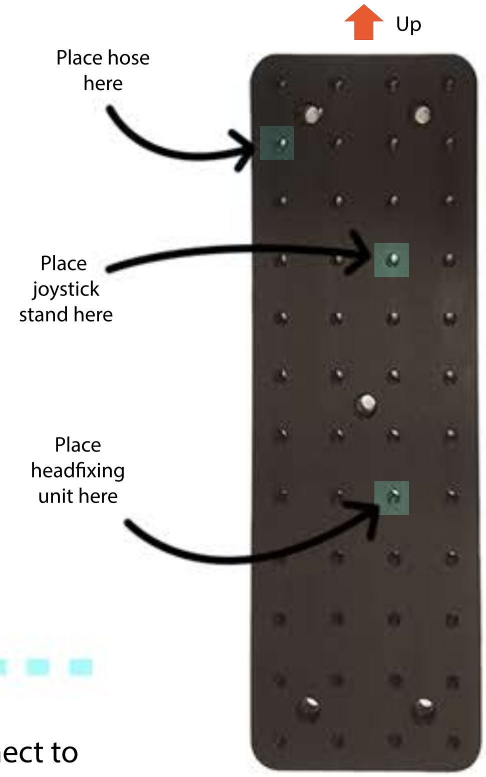

Goal: Build an Arduino controlled water delivery system and connect to joystick and headfixing unit

1. Using online instructions, create an Arduino controlled solenoid. Instructions found at:

https://www.instructables.com/id/Controlling-solenoids-with-arduino/

Look at the schematic wiring diagram to check that current is flowing through the entire circuit (ex: resistor and diodes are pointing the correct way), and that the solenoid is properly connected.

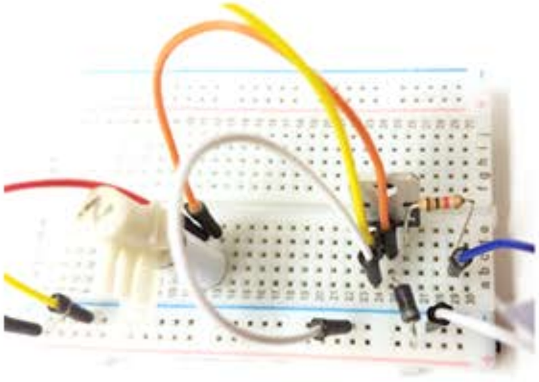

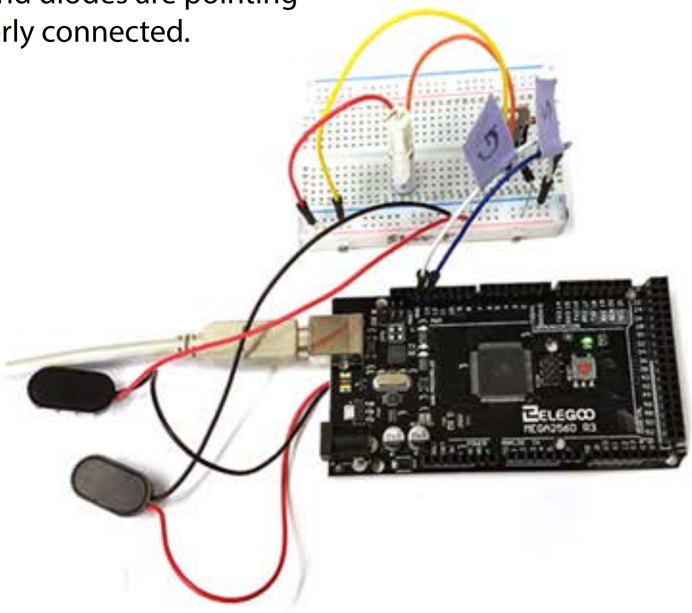

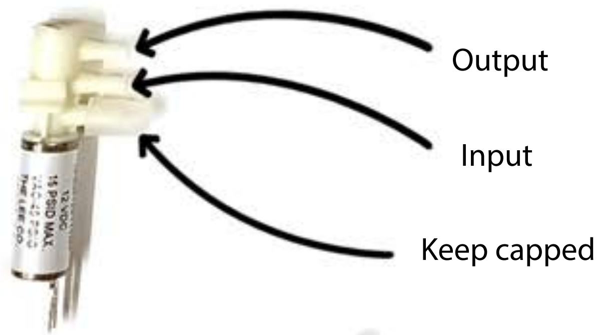

2. Once ciruit is complete, attach lick spout tubing to the output valve of the solenoid. Attach other length of Tygon tubing to the input valve. If using the 3-Port HDI solenoid, leave the third valve capped.

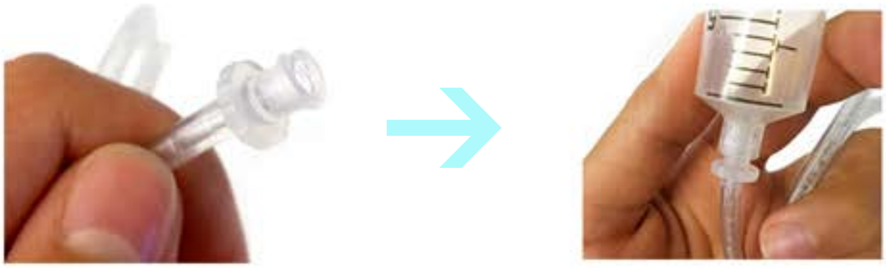

3. Push unconnected end of input valve tubing over Luer loc screw, and screw into base of Luer lock 30ml syringe.

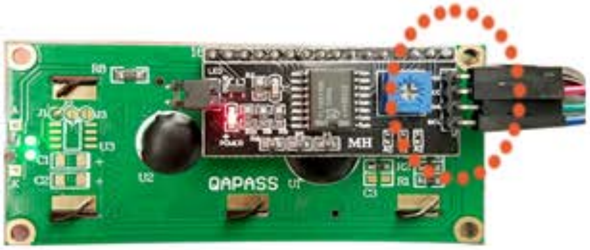

4. Connect LCD screen to the Arduino board, so that mouse progress can be tracked in real time. Using the back of the screen as a guide, connect the screen to the Arduino board through ports SCL, SDA, Ground, and 5V with four male/male jumper cables.

5. For final assembly of system, tape tube connected syringe on to a wall near where the rig will be kept. Arrange Arduino board and electrical breadboard onto optical bread-board with headfixing unit, to create a con-tained, modular training system. Secure down pieces with tape or twist ties.

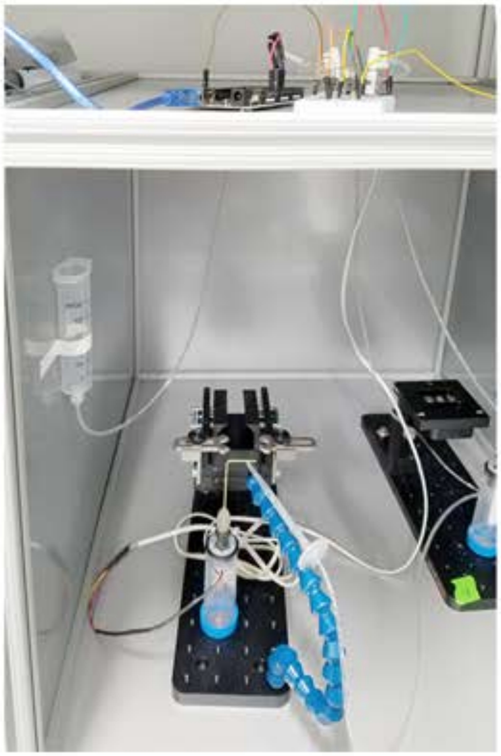

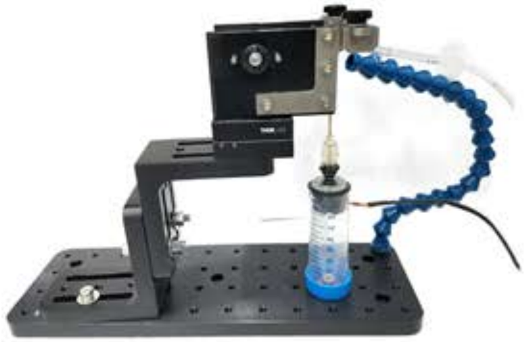

Notes on Data Collection

Because neither the Arduino IDE nor board store data, joystick information must be held in alternate programs like Processing, or on additional hardware, like micro SD cards. Through both collection methods, .csv files containing desired information can be generated and read through data processing software such as MATLAB.

Processing:
1. Set up a Processing script to write files to a certain folder path.
2. Make sure board is directly connected to computer, by way of the A/B USB cable.
3. Select the correct port in Arduino, from the pull down menu path: Tools>Port
4. To record data, run the Processing script (play button). Check that the file is actively writing to the desired folder. If it is not, stop the script, unplug/re-plug the A/B cable, and try again.

Micro SD:
1. Set up MATLAB script to pull files off of micro SD card.
2. Attach card reader to Arduino board, based on inputs (CS, SCK, MOSI, MISO, VCC, GND) on the back of the device with 6 Female/Male jumper cables.
3. When recording data, make sure micro SD card has "clicked" into place in the reader. To stop recording, take the SD card out of the reader.

Software links:
Arduino IDE – https://www.arduino.cc/en/main/software
Processing – https://processing.org/download/

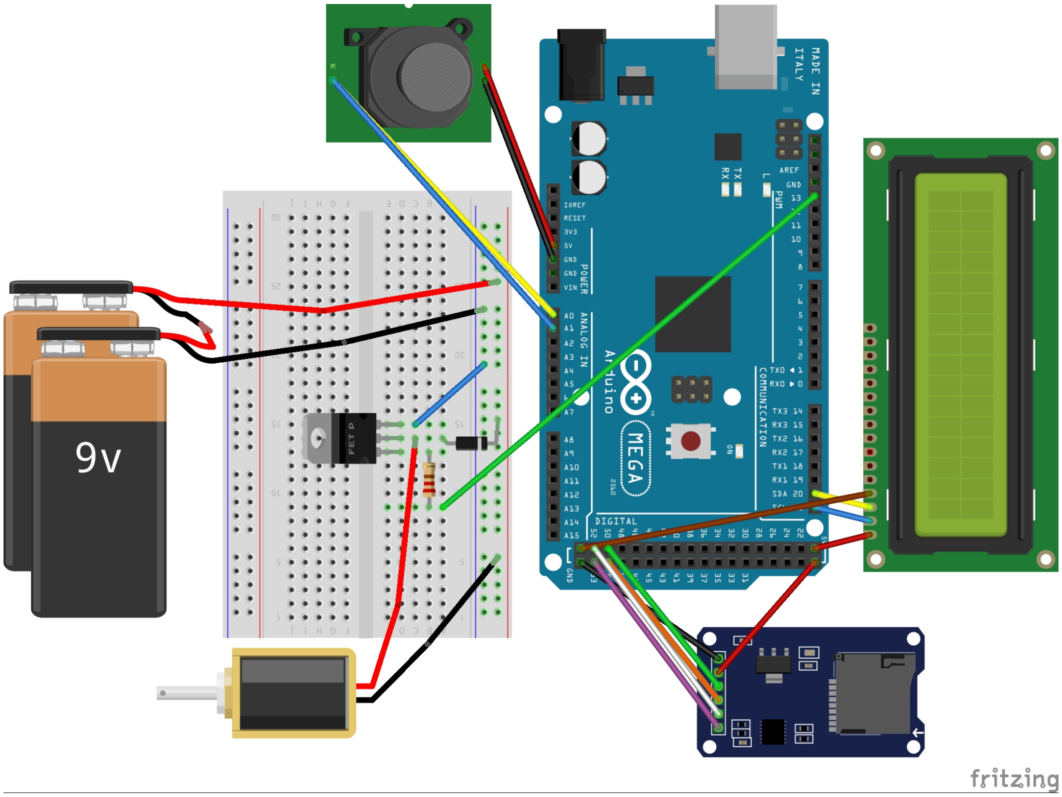

Materials Order Form

Base Lab Tools:

Item	Catalog Number	Description	Price
¼ -20 Stainless Steel Screw Kit	SKT25	Screw kit including ¼– 20 Cap Screw, Set Screws, Nuts, Washers	$49.00
Optical Breadboard	SAB0412-D	4" x 12" x 1/2" Solid Aluminum Optical Breadboard, Black Anodized	$65.00
Right Angle Mounting Bracket	ABS002	2 in Right Angle Mounting Bracket, Narrow Slotted (1)	$39.00

McMaster Carr:

Item	Catalog Number	Description	Price
0.34" ID, 0.875" OD Washer	93490A030	Bronze Washer for 5/16" Screw Size, 0.34" ID, 0.875" OD (pack of 10)	$5.70
1/16" ID, 1/8" OD Tubing	6516T11	Tygon PVC Clear Tubing 1/16" ID, 1/8" OD, 25 ft length	$5.75
1/16" Brass Wire	8859K511	Ultra-Formable 260 Brass 1 Foot Long Rod, 1/16" Diameter (pack of 3)	$2.03
Loc-Line	10095K97	Loc-Line Coolant Hose 1/4" Trade Size, Female x Male, 5 Feet Long	$28.20
Male Luer Lock 1/8" ID Hose Barb	51525K291	Plastic Quick-Turn Tube Coupling Sockets, for 1/16" Barbed Tube ID, Polypropylene (pack of 10)	$5.24

Thorlabs:

Item	Catalog Number	Description	Price
Kinematic Base	KB2X2	2" x 2" Kinematic Base, Top and Bottom Plates, 1/4"-20 Mounting	$83.39

Ruffy Controls:

Item	Catalog Number	Description	Price
Miniature 2 Axis Hall Effect Joystick	TS1-1-R-R-1-BK	TS1, Stepped Cap, Round Limiter, rear Mount, 0-5v, Black	$75.00

Items from Other Retailors:

Item	Name on Site	Website	Price
3 – Port HDI Solenoid	1303	https://sanworks.io/shop/viewproduct?productID=1303	$74.00
30 ml Syringes	30 mL Syringe Luer-Lok Tip, Box of 56	https://www.vitalitymedical.com/30-ml-syringes-without-needle.html	$25.84
50 ml Conical Tubes	14-432-22, Case of 500	https://www.fishersci.com/shop/products/falcon-50ml-conicalcentrifuge-tubes-2/p-193321	$379.25
Battery Clip Connector	9V Volt Clip on Type Battery Snap Connector Lead Wire Plastic Head, 10 Pieces	https://www.amazon.com/Battery-Connector-Plastic-Atomic-Market/dp/B00IDHZ5FM	$6.99

1K Diodes	1N5408 Rectifier Diode 3A 1000V	https://www.amazon.com/Parts-Express-1N5408-Rectifier-Diode/dp/B0009XSN02	$6.91
Electrical Breadboard	BB400 Solderless Plug-in BreadBoard, 400 tie-points, 4 power rails, 3.3 x 2.2 x 0.3in (84 x 55 x 9mm)	https://www.amazon.com/BB400-Solderless-Plug-BreadBoard-tiepoints/ dp/B0040Z1ERO	$5.90
Elegoo Board	Elegoo EL-CB-001 UNO R3 Board ATmega328P ATMEGA16U2 with USB Cable for Arduino	https://www.amazon.com/Elegoo-EL-CB-001-ATmega328PATMEGA16U2-Arduino/dp/B01EWOE0UU	$10.86
Heat Shrink Tubing, 1/8"	Polyolefin 2:1 Heat Shrink Tubing	https://www.amazon.com/Polyolefin-Heat-Shrink-Tubing-Inch/dp/B01G5RQ3KW	$16.99
Jumper Cables	40pin Male to Female, 40pin Male to Male, 40pin Female to Female Breadboard Jumper Wire Ribbon Dupont Cables Kit, 120 Pieces	https://www.amazon.com/COMeap-120pcs-Female-Breadboard-Jumper/dp/B01MU0IMFF/ref=sr_1_4?s=industrial&ie=UTF8&qid=1538699670&sr=1-4&keywords=male+male+jumper+cables	$7.99
LCD Screen	LGDehome IIC/I2C/TWI LCD 1602 16x2 Serial Interface Adapter Module Blue Backlight for Arduino UNO R3 MEGA2560 (2 pack)	https://www.amazon.com/LGDehome-Interface-Adapter-Backlight-MEGA2560/dp/B0711WLVP9	$9.59
Micro SD Card	Micro SD Card 32GB,AUAMOZ Micro SDHC Class 10 UHS-I High Speed Memory Card for Phone,Tablet and PCs - with Adapter (2 Pack)	https://www.amazon.com/gp/product/B07DGHCFSM/ref=oh_aui_search_asin_title?ie=UTF8&psc=1	$16.12
Micro SD Card Reader for Arduino	SenMod 5PCS Micro SD Card Micro SDHC Mini TF Card Adapter Reader Module for Arduino	https://www.amazon.com/gp/product/B01JYNEX56/ref=ppx_yo_dt_b_asin_title_o00__o00_s00?ie=UTF8&psc=1	$8.29
RJ11 Telephone Cable	C2G/Cables to Go 02970 RJ11 Modular Telephone Cable, Silver (7 Feet, 2.13 Meters)	https://www.amazon.com/C2GCables- Modular-Telephone-Silver/dp/B00006HSK6	$3.47
60 V Transistors	Major Brands TIP120. Transistor, Darlington, NPN, 60 Volt, 5Amp, 3-Pin, 3+ Tab, TO-220, AmpB, Rail, Pack of 15	https://www.amazon.com/Major-Brands-TIP120-Transistor-Darlington/dp/B00B888622/ref=lp_306910011_1_7?s=industrial&ie=UTF8&qid=1538700353&sr=1-7	$5.99

Approximate Cost of One Setup: $440
