# Supplementary figures and images for: Open-Source Joystick Manipulandum for Decision-Making, Reaching, and Motor Control Studies in Mice
Source: eNeuro. 2020 Mar 24;7(2):ENEURO.0523-19.2020. doi: 10.1523/ENEURO.0523-19.2020 (PMC7131984; doi:10.1523/ENEURO.0523-19.2020)

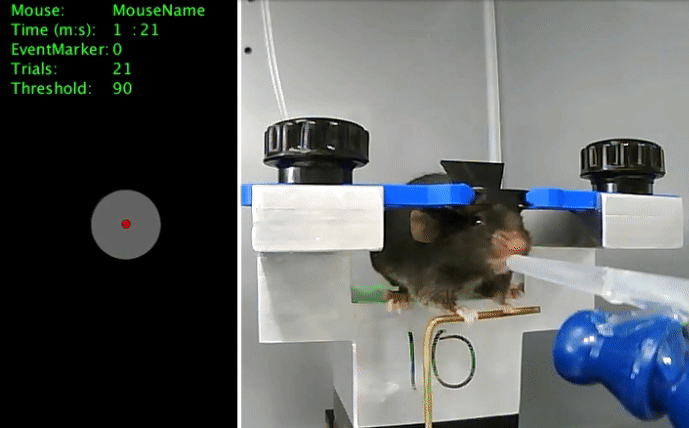

Supplement: Extended Data 2 — Online and offline joystick code. Online and offline code can be found at the Yttri Lab GitHub (https://github.com/YttriLab/Joystick). “Arduino Code” contains sketches to run the basic center-out reaching task, the VAO task, the reaction time task, and the directional-dependent two-armed bandit task. All tasks are capable of tracking real-time joystick position and allow for experimenter defined control of task parameters. The folder also includes code to flush fluid delivery lines for cleaning. Supplied in “Processing Code” is a sketch that can be used to visualize real-time joystick position as well as task performance and variables for the basic center-out reaching task. “MATLAB Code” contains the main offline analysis code (JSAnalysis.m) for the basic center-out reaching task, which collects data pertaining to task performance as well as reach kinematics. This folder also includes accessory analysis functions and a function (SavemicroSDData.m) to save and name data collected from the joystick. Download Extended Data 2, ZIP file. [file enu-eN-OTM-0523-19-s04.zip › JS-Manual-master/Demo/JSGithubDemo.gif]
